# Analysis of gaze patterns during facade inspection to understand inspector sense-making processes

**DOI:** 10.1038/s41598-023-29950-w

**Published:** 2023-02-20

**Authors:** Muhammad Rakeh Saleem, Robert Mayne, Rebecca Napolitano

**Affiliations:** 1 Department of Architectural Engineering, The Pennsylvania State University, University Park, PA 16802 USA; 2 Department of Mathematics, Chariho Regional High School, Richmond, RI 02894 USA

**Keywords:** Civil engineering, Human behaviour, Statistics

## Abstract

This work seeks to capture how an expert interacts with a structure during a facade inspection so that more detailed and situationally-aware inspections can be done with autonomous robots in the future. Eye tracking maps where an inspector is looking during a structural inspection, and it recognizes implicit human attention. Experiments were performed on a facade during a damage assessment to analyze key, visually-based features that are important for understanding human-infrastructure interaction during the process. For data collection and analysis, experiments were conducted to assess an inspector’s behavioral changes while assessing a real structure. These eye tracking features provided the basis for the inspector’s intent prediction and were used to understand how humans interact with the structure during the inspection processes. This method will facilitate information-sharing and decision-making during the inspection processes for collaborative human-robot teams; thus, it will enable unmanned aerial vehicle (UAV) for future building inspection through artificial intelligence support.

## Introduction

Structural inspections are critical for routine maintenance and therefore have been codified into laws and regulation^1,2^. Presently, there are three inspection modalities: (1) human-driven visual inspection, (2) pre-programmed flight path-based UAV-driven visual inspection, and (3) human-guided remote UAV inspection. In human-driven visual inspection, inspectors are sent to walk around structures and take notes; while this requires minimum technology, this method can introduce bias and safety concerns. Entering the vicinity of damaged structures for inspections especially after a disaster, can pose a serious safety risk due to the unknown structural integrity^3^. Additionally, the quality and consistency of visual inspection (VI) greatly depend on the inspector’s training experience, equipment, and bias. In UAV-driven visual inspection, UAVs are given condition-agnostic flight paths to capture images of a structure. While this requires low levels of technology and can be safer for inspections, this method lacks decision-making skills for dynamic imaging such as zooming^4^. The remote inspector relies on the camera feed and the flight mission to perform the inspection. Once the UAV starts its mission, there is no reactive control except to abort the mission before the flight path is completed. In human-guided remote UAV inspection, a remote inspector can pilot the drone. While this is safer for an inspector, this requires more drone piloting expertise and there is no critical thinking or sense-making shared between the inspector and the UAV^5^.

In comparison, both the first and third modalities can be reactive, meaning decision making can be done based on the detected damage; however, the inspector’s knowledge depends on their expertise in visual inspection. The second modality prohibits reactive decision-making and restricts following a pre-defined flight path once the mission is started. Although VI is currently the predominant inspection procedure and is followed by many practitioners, this approach has several limitations. In much research, it has been found that VI by itself is unreliable and not consistently efficacious for identifying repair priorities^6^. Currently, nine U.S. cities have mandated ordinances for periodic inspections of their buildings’ facades, and many other cities are adopting these regulations^7^. Although the standards and guidelines for facade inspection are well-developed^8^, these inspections are not done as frequently as they should, and the average frequency of facade inspection is five years. Additionally, the frequency of these inspections also depends on (1) building height, (2) number of stories, (3) building age, and (4) topography. These inspections cost time, labor, and safety concerns as well as they can introduce subjectivity in the results^9^. Additionally, the quality and consistency of VI greatly depend on the inspector’s training experience, equipment, and bias^3^.

The ability of an autonomous system to understand something about a human’s intent is vital to the success of many systems that involve both humans and autonomous agents. Eye movements can inform what a person is paying attention to, what they spend most of their time on, and how they move between different elements in a visual scene^10^. While many researchers have examined the connection between human actions and implicit behavior^11^, the abilities to infer human thought and the decision-making processes have not been thoroughly investigated in terms of human-structure interaction. While machines are very competent at specified problem-solving, many are brittle when they confront unusual data^12^ and cannot adapt to flexible environments. In the context of an inspection, it is essential to know if the person is searching for a particular damage or considering a specific location versus scanning the entirety of the structure for anomalies^13^. For example, when a human inspector examines a large facade for damage, they look at the structure dynamically to identify potential defects. While this is occurring, the inspector is not aware of the electrical signals contracting their eye muscles (squinting) and neck (turning)^14^. The human brain actively transmits signals to the sensory system through neurons and compensates for new information using feedback control and commonsense reasoning. While this is something a human can do within the blink of an eye, we must understand the metrics that are responsible for sense-making and/or decision-making if we are to understand this process and its relationship to human biases and/or controlling intelligent, automated systems in the future.

Recently, there has been significant advancement in eye tracking and cognitive assessment technology; it is becoming more affordable, accurate, and easy to use^15^. Many companies and researchers have focused on this idea of cognitive thought-process and how it compliments decision-making for many diverse applications. Many researchers use AR technologies for human-infrastructure interaction^16,17^ and structural health monitoring^18^ to quantify structural conditions with more objective data. The use of a convolutional neural network (CNN) for facade damage detection and structural inspection is a well-known technique. However, training a CNN requires a large amount of data, high computing capability, and real-world data, yet it fails to capture expert knowledge, and there is no reasoning for damage assessment and inspection from a documentation point of view. Further, most of the work is done in simulated environments without capturing real-world scenarios. However, eye tracking can be used to study eye movement and can accurately inform where an inspector is looking and paying close attention for detailed damage assessment. This can inform reactive decision-making and is helpful for preventive maintenance, incorporating humans and machines for human-building interactions. Considering these advancements, the objective of this study is to examine human eye gaze patterns and observe how the human brain compensates for any new/unexpected information during a facade inspection using eye tracking-enabled feedback control and sense-making. Eventually, this information can inform intelligent, autonomous facade inspection agents for structural inspection in the future studies. The focus of this study will remain on analysis of human gaze patterns and eye tracking metrics to identify eye movements and inspector’s abilities to assess and diagnose the structure.

## Methods

### Participants

Eleven participants (seven males and four females) were recruited from the architectural engineering (AE) department at The Pennsylvania State University (PSU) to participate in this study. All participants had normal or corrected-to-normal vision, and they ranged in age from 26 to 34 years (mean age = 28, SD = 2.7). One participant was excluded from the final sample because of their eye vision. Additionally, participants had some previous knowledge of structural inspection and engineering. The study follow the ethical guidelines of Human Subjects Research and was approved by the Institutional Review Board (IRB) for Human Research Protection Program (HRPP) of The Pennsylvania State University (STUDY00016625). Informed consent was given by the participants before the start of the experiment.

### Apparatus

Eye movement data were recorded using Tobii Pro Glasses 3^19^ wearable eye tracking equipment developed by Tobii Technology (100 Hz sampling rate; $1920 \times 1080$ resolution @ 25 fps; and 4 eye movement cameras). The eye movement of the subject is tracked by pupil corneal reflection and binocular dark pupil acquisition technology, which has wireless real-time observation function and supports slip compensation. Pro Glasses 3 has a proprietary SDK that allows one-point calibration of gaze patterns. According to their manual guide^20^, the eye tracker firmware needs to adapt the algorithms to the person wearing the wearable eye trackers for high accuracy and precision. During the calibration process, the participant must wear the head unit and focus their gaze at the center of the calibration card, holding it at about arm’s length of 0.5–1 m. Once the gaze point and calibration marker are stable, a calibration completion prompt appears on the screen. In this experiment, Tobii’s Glasses Controller software was used for data acquisition provided by the manufacturer (Tobii Technology) which uses one-point method for eye movement calibration, and offers live-view scene of the observation. For post-processing and analysis, Tobii Pro Lab software^20^ provides a visual and functional user interface for complex experiment design, observe and qualitatively analyze individual recordings, and aggregate data for quantitative analysis and visualization.

### Procedure

The purpose of this study was to analyze human gaze patterns and determine eye tracking metrics that were important for decision-making while performing a facade inspection. To this end, we have performed experimental studies on participants to collect preliminary data about the intuition of human interaction within a damaged infrastructure environment. The experiment was conducted in an outdoor environment on two, three-story, steel-frame buildings with masonry infill and veneer in central Pennsylvania^21^. The buildings under inspection have minor cracks, surface stains, weathered stone, missing mortar, bio-growth, and peeling paint etc,.

#### Design experiment

**Figure 1:**
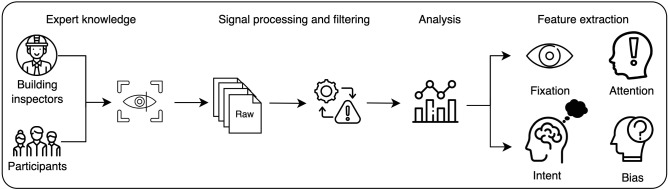
An overall framework for Eye tracking based infrastructure inspection.

Participants were given an experimental prompt (refer to appendix I) before the start of experiment and were asked to perform the task of structural assessment accordingly. The first step was to capture expert knowledge of participants by recording their eye movements and gaze data while looking freely at a structure in their surroundings. These eye movements can be classified into fixations and saccades; fixations are when eyes are stable and stationary looking at a particular region of interest in the scene, and saccades are the rapid eye movements that occur between fixations when a person looks from one fixation to another. Once the data is collected, feature extraction was carried out to capture eye tracking metrics and understand their importance. These eye tracking metrics will form the basis of understanding gaze patterns for structural inspection (see Fig. 1).

#### Data analysis

For qualitative analysis, heat maps and gaze plots provide fast and accurate data visualizations that are important in understanding aspects of visual behavior. One example of a “Heat Map” and “Gaze plot is shown in Supplementary Fig. 1 for detail. The heat map describes the overall distribution of a participant’s vision over a particular stimulus. The areas with red color indicate the high intensity of a participant’s fixations compared to low-intensity areas indicated by light green. Heat maps can effectively document visual attention in a scene and consider all the fixations of a participant; this can be used to understand decision-making processes. Similarly, individual gaze plots vary based on a specific person’s fixations and scanning strategies which correspond to their gaze trail and involuntary, saccadic eye movement. The longer the fixation duration, the larger the circle diameter; the number inside the circle indicates the order of where the participants looked.

Several studies^22–24^ discuss how bias can occur for various reasons, regardless of a person’s intent. These biases can significantly affect the decision-making of an inspector while enacting engineering judgment. If a person is looking at an image or a scene in the real world, they will have a bias of some sort, which depends on their perception of importance. In addition, bias can be introduced in eye tracking measurements for various reasons and should be quantified to minimize any adverse effects. These biases can appear while inspecting and affect decision-making^25^. The most prominent biases that can manifest in an inspection are attentional bias^26^, fixation bias^27^, cognitive bias^28^, implicit bias^29^, and negativity bias^30^.

#### Data processing

Since viewing patterns can vary widely among participants, in disparate research fields, and even in a visual scene, it is impossible to determine the optimal parametric values for all use cases in eye tracking research. Thus, this work seeks to understand the relationship between parameters and extracted features. Velocity-threshold identification (I-VT) fixations, attention, and raw filters^31^ were tuned for fixation classification. One challenge in visual building inspection is that head and body movements can affect data collection and the efficacy of certain measurements. Thus, we must choose the attention and/or fixation filter that fits the experimental design requirements (see appendix III, Supplementary Figs. 2, 3 and 4). Many different algorithms have been used to identify eye movements such as saccades or smooth pursuits^32^, and depending on the kind of eye movement that is of interest, different classification algorithms are more or less suitable. In this study, we have selected an attention filter as we are more interested in understanding where a person’s gaze is fixated and what grabs their focus while assessing a structure dynamically. The I-VT attention classification algorithm is a velocity-based classification algorithm^33^ based on the velocity of the directional shifts of the eye measured in visual degrees per second ($^{\circ }/s$). If it is above the pre-defined threshold for the I-VT filter, it is classified as a saccade, and if it is below, it is seen as part of a fixation.

### Statistical modeling methods

#### Standardization of variables

The eye tracking metrics contain time and count information and must be converted into a form where proportional comparison can be easily made. For this, time/area was a robust standard and the dimensions and proportions are calculated base on the test images in pro lab that were annotated for specific damage. The approximated areas were calculated based on the image resolution and measuring scale.

#### Method for analyzing surrounding versus damaged regions

The eye tracking metrics under consideration (i.e., fixation duration, fixation count, visit duration, and visit count) were compared to determine if there is a difference in the means between surrounding and damaged areas. This informs how the surrounding and damage regions are different statistically which will prove our null hypothesis. The duration and numbers of fixations and visits were added together for all AOIs then divided by the sum of all areas of the AOIs. Since each recording represents one of the ten participants and the experience of the participant may have an influence on the metric per AOI, therefore, these data are considered paired and a paired t-test will be conducted. Since these data are paired, we are interested in the differences between the two means (difference = surrounding - damaged) i.e., $H_{o}: \mu _{difference} = 0$ and $H_{A}: \mu _{difference} > 0$. Our null hypothesis was that there is no significant difference among surrounding and damage areas, and that needs to be nullified to test that participants paid more attention on damage regions compared to surroundings while scanning and detecting the facade damage.

To perform a paired t-test, the condition of normality must be checked using either a Normal q-q (Quartile-Quartile) plot or a Shapiro-wilks test. The Shapiro-wilks test is more formal test to check the normalcy condition while a q-q plot is an informal test of normality that can give you some insight into the nature of the data qualitatively. Since it is only a visual plot, it can be rather subjective. For a more definitive normalcy check, we opt for Shapiro-Wilk test to calculate *“W”* (Note: No adjustments were made to the data and the test results showed they are normally distributed).

#### Method for analyzing individual damage type

Since each observation is independent from the other, and the AOIs relative to the participant are not independent based upon the experience of the participant, a repeated measures one-way ANOVA test is performed. The condition of hypothesis here is to check if there is difference among different damage types for different participants and our null hypothesis would be, $H_{o}; \mu _{BG} = \mu _{crack} = \mu _{MM} = .... = \mu _{WS}$ and alternative hypothesis would be, $H_{A}; \mu _{BG} \ne \mu _{crack} \ne \mu _{MM} \ne .... \ne \mu _{WS}$.

As the data did not pass the normality test and therefore logarithmic transformation was applied for one-way ANOVA analysis. Additionally, we used Bonferroni’s correction when analyzing the individual AOIs to the surrounding areas to reduce false-positive in our results for multiple pairwise testing performed on a single data set.

## Results

### Experimental trial 1

#### Heat map and Gaze plot interpretation

**Figure 2:**
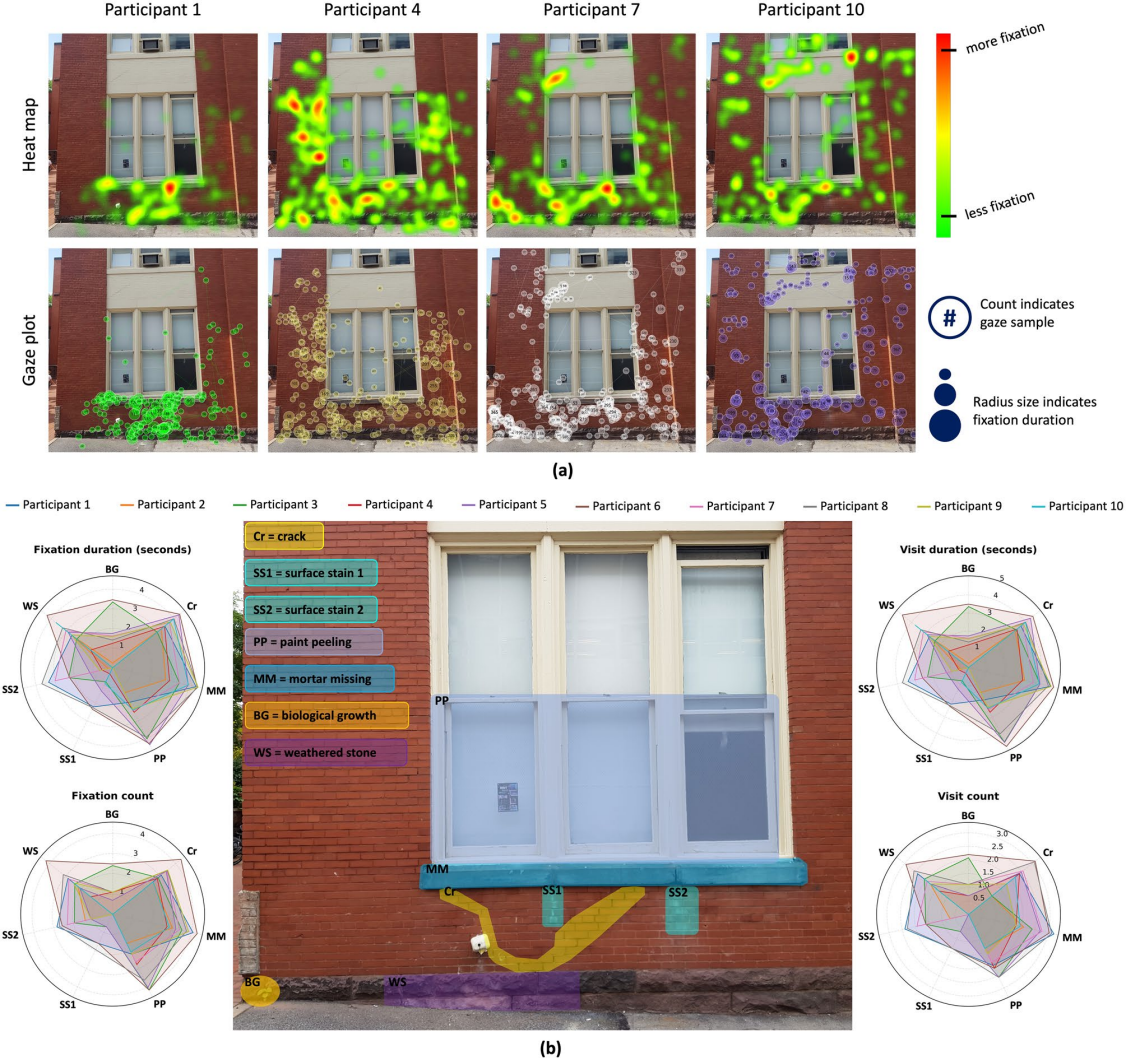
Results of participants data for Building 1 (**a**) Comparison among heat maps and gaze plots of participants ($\textit{n} = 4$ in visual for simplicity; $n_{\textit{total}}$ = 10), (**b**) Comparison of participant’s eye tracking movements based on damage typology i.e., fixation duration(seconds), fixation count, visit duration (seconds), visit count; legend identify 10 candidates participated in the experiment.

The first experiment was performed on the facade of Building 1. The test structure is shown in Fig. 2a, indicating different types of damage such as cracks, surface stain, biological growth, missing mortar, peeling-off paint, and weathered stone. The damage was caused due to environmental factors and lack of maintenance resulting in wear and tear of the building facade. Fig. 2a shows a comparison of heat maps and gaze plots for different participants ($n=4$ for simplicity, $n_{total}=10$; see Supplementary Figs. 5-24, for detail) and provides qualitative information about how each participant viewed different elements of the building facade. Each heat map shows the fixation patterns for individual participants, which help understand their gaze; here, red indicates long fixation periods, and green represents short fixation periods. Similarly, the gaze plot (see Fig. 2a) shows the participant’s eye patterns and gaze trail as they move their eyes from one element to another. The numbers in the circles represent the order of the gaze from one fixation to another. It can be observed in Fig. 2a that the gaze plot of the participants varies, which could indicate differences in their diagnostic reasoning capabilities and understanding of the test stimulus while looking for other damage.

Based on the results of participant’s heat map and gaze plot as shown in Fig. 2a (see Supplementary Figs. 5-24 for detail), P1’s heat map and gaze plot indicate that they looked at the bottom center of the building facade and focused mainly on crack regions. Additionally, they had very few fixations on the top portion of the facade. P4’s heat map and gaze plot showed an overall distribution of fixation with emphasis on masonry bricks and foundation stones. Also, they spend some time fixating crack damage and weathered stone damage. P7’s heat map indicates fixation on masonry cracks, biological growth, and out-of-plane bricks on the structure with fewer fixations towards the right side of the facade. The gaze plot also indicates the human gaze with a longer fixation duration on cracks in masonry and biological growth. P10’s heat map shows their overall focus on the facade structure with well distributed gaze points across the facade completely. They spend more fixations on crack and out-of-plane brick damage, with fewer fixations on different parts of the structure. The gaze trail indicates the element-wise assessment from the bottom center to the bottom right and then the top portion of the structure.

#### Selected AOI metric analysis

For more rigorous analysis, fixation and visit metrics were analyzed for individual participants based on the damage typology, and the results are shown in Fig. 2b. The building facade (see Fig. 2b) was labeled based on the different damage typologies such as crack, missing mortar in masonry joints, surface stains, peeling paint, weathered stone at the foundation, biological growth, etc. These AOIs were segmented as potential damage regions for facade inspection. As described in the previous section, fixation and visit metrics were analyzed in detail, corresponding to each participant’s damage AOIs, and the analytical results are shown in Fig. 2b. For the seven damage regions, Fig. 2b shows the total fixation duration and fixation count (on the left), and Fig. 2b shows the entire visit duration and visit count (on the right), respectively, of all ten participants for different damaged locations on the building facade.

Based on the collected data, all the participants identified crack, missing mortar, and paint peeling-off damage with comparative fixation and visit duration with fewer visits from P2 and P3. Weathered stone and surface stain 1 damage were not analyzed for too long by most participants and they have minor visits on them. Biological growth and surface stain 2 damage were not analyzed by half of the participants and this could be due to the fact that surface stain damage look alike and they did not spent longer fixation duration on those damage. On average, participant 6 spent more time fixating for damage assessment while participant 2 spent less time on most damage compared to the rest of the group. Additionally, participants’ 6, 8, and 10 have higher visit counts among other showing their frequent visits between different damage typologies. Some participants have less or no fixation and visit count, however they have fixation duration on those damage and therefore we can not rule-out by the fact that they miss or over-look certain damage.

### Experimental trial 2

#### Heat map and Gaze plot interpretation

**Figure 3:**
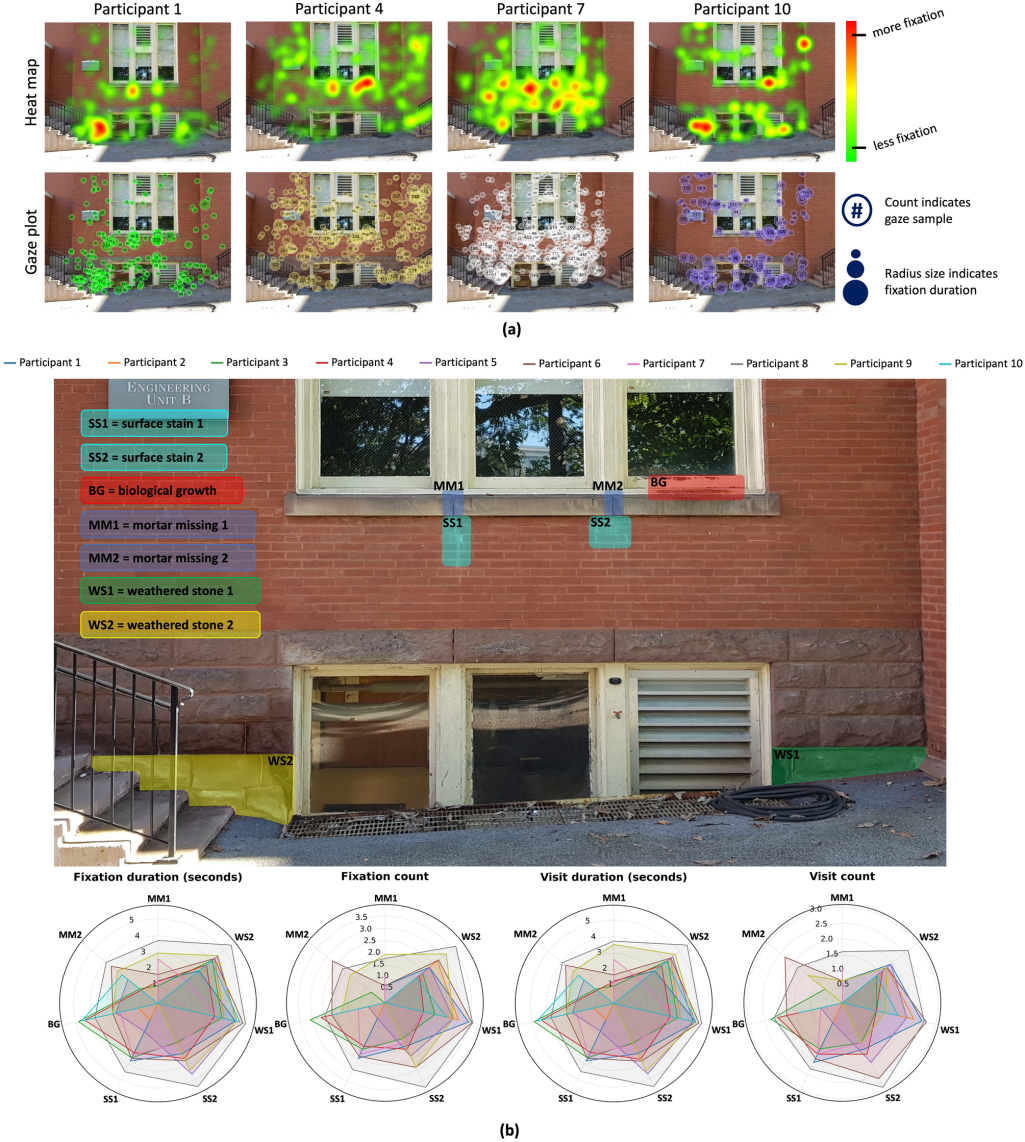
Results of participants data for Building 2 (**a**) Comparison among heat maps and gaze plots of participants ($\textit{n} = 4$ in visual for simplicity; $n_{\textit{total}}$ = 10), (**b**) Comparison of participant’s eye tracking movements based on damage typology i.e., fixation duration(seconds), fixation count, visit duration (seconds), visit count; legend identify 10 candidates participated in the experiment.

Unlike Building 1, Building 2 facade does not have crack damage but entails severely weathered stone, indicating degradation of the building foundation. The damage typologies for Building 2 consisted of missing mortar in the window frame, surface staining due to water infiltration, biological growth, and weathered stone at the foundation blocks as shown in Fig. 3a. Participants were given the same prompt to read and follow before beginning the inspection (see Supplementary Figs. 25-44 for detail). Fig. 3a compares heat maps and gaze plots for four participants ($n=4$ for simplicity, $n_{total}=10$; see Supplementary Figs. 25-44 for detail). Similar to Building 1, the heat maps in Fig. 3a shows the fixation patterns for individual participants, which helps in understanding their gaze; the gaze plot shows the participant’s fixation locations and gaze trail as they move their eye from one element of the building facade to another. Fig. 3a highlights the difference in the participants’ heat maps and gaze plots, which could mean differences in their diagnostic reasoning capabilities and how they recognize and perceive a test structure while assessing potential damage.

Considering the data for Building 2 based on Fig. 3a, P1, P4 and P7 scanned the structure holistically while P10 looked at certain regions of the structure. P1’s heat map indicates significant fixations on the bottom left and below the window frame of the building facade. This is evidenced by the gaze plot with larger fixations circles and small gaze trail for bottom left and smaller fixation duration for the other regions of the structure. P4 looked at the biological growth and missing mortar damage with longer fixations and they spend comparatively less time on foundation stones. The gaze plot indicates larger fixation circles with small gaze trail meaning they analyzed the structure in detail. The heat map of P7 indicate larger fixations on the lower portion of the building facade with more duration time on masonry facade below the window. They also have higher fixations at the left weathered stones in the foundation which is also evidenced by the gaze plot. P10’s heat map showed they analyzed the foundation stones with more details and the window frame region with biological growth and weathered stone damage. However, in general they spent less time on the facade damage compared to the other four participants as indicated in the Fig. 3a.

#### Selected AOI metric analysis

Similar to previous section, for more detailed analysis, fixation and visit metrics were also analyzed for Building 2 individual participants, as shown in Fig. 3b. The building facade was annotated with several damage and the result of these metrics for different participants in also shown 3b. For Building 2, we have marked surface stain below window frame, missing mortar in the window frame, biological growth present on the right side of the window, and damage in the foundation stone on the end next to staircase.

Based on the eye tracking metrics for the collected data, P8 spent longer fixation duration on average on all the damage regions while P2 only focused on weathered stone and biological growth damage with smaller fixation duration. Foundation stones are one of the high fixated areas of the facade and is assessed by all the participants for longer fixation. This could be due to the fact that this damage is more obvious compared to other because of the damage surface area. Furthermore, fixation and visit count also indicate weathered stone and surface stain damage are the areas with more frequent fixations indicating participants back-and-forth eye movements and little-to-no attention by missing mortar damage. From these results, it is concluded that different types of damage can associate different damage severity and there is significant difference among the types of damage to different participants.

### AOI versus non-AOI based analysis

**Figure 4:**
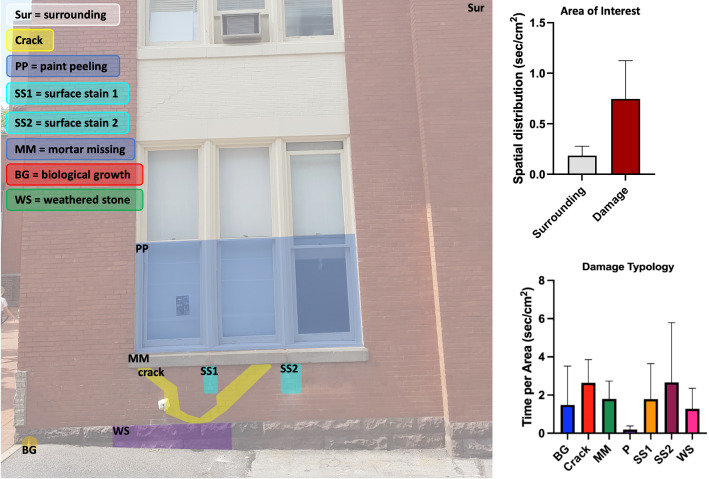
Total fixation time on different damage areas of interest (AOIs) during visual inspection; (left) location and size of AOIs (bottom-left) total fixation duration of damage to surrounding areas; (top-right) comparison of participant’s overall spatial distribution of surrounding vs. damage areas;(bottom-right) breakdown of total spatial distribution to individual damage type fixations for all participants.

For statistical analysis of eye tracking metrics, as explained in statistical modeling section, based on damage and non-damage regions of the facade structure, participant’s total dwell time for damage AOIs to surrounding region was considered. The marked regions for various damage typologies were considered as damage region versus the rest of the facade structure as surrounding. Fig. 4(left) showed the classification of structural facade into damage and non-damage regions while Fig. 4 (Area of Interest) indicates the spatial distribution of all participants gaze. To compare surrounding region to damage region, paired t-test was performed to determine if there was significant difference statistically among damage and surrounding areas. The results showed damage and surrounding areas were statistically different ($\textit{p} < 0.05; t = 5.315, df = 9$) and Fig. 4 (area of interest) indicate the difference among the two categories for fixation duration metric. Table. 1 showed the results for all four eye tracking metrics and how notable their difference were for damage to non-damage(surrounding) regions. Another assumption was that participants’ spent more time on damage areas compared to surrounding despite the fact that the surface area of damage regions was very small. To understand this better, spatial distribution of participants was analyzed using *time/area* metric as shown in top right illustration. This tells that participants’ spent almost 3 times more time looking at damage regions of the facade structure which is evidence in our assumption that participants (inspectors) spent more time on damage regions to non-damage region while doing structural assessment.

**Table 1:** Descriptive statistics of eye tracking metrics for spatial distribution of surrounding vs. damage regions where$\textit{p} < 0.05$.

	Building-1				Building-2			
	Mean	Std. deviation	*t*-value	*p* value	Mean	Std. deviation	*t*-value	*p* value
Fixation duration	0.5605	0.3334	5.315	0.0002	1.208	0.2739	13.95	<0.0001
Fixation count	1.710	1.366	3.959	0.0017	1.083	0.2523	13.57	<0.0001
Visit duration	0.5714	0.4804	3.762	0.0022	1.051	0.2796	11.89	<0.0001
Visit count	0.8881	0.4255	6.599	<0.0001	1.770	0.0848	66.05	<0.0001

Further, damage regions were broken down into individual damage regions. To determine if the mean fixation time was the same for the surrounding area and the individual damage regions, a repeated measures one-way ANOVA test was conducted. The results showed that the means were not equal $(F (3.768, 31.22) = 26.30, p < 0.0001)$. As shown in the bottom-right of Fig. 4 (damage typology), crack and surface stain damage receive more attention and longer fixation time from all participants followed by missing mortar in masonry bricks. The least amount of attention was paid to the paint peeling of the window frame as this could be justified by the fact that paint damage is not considered structural damage. To determine if differences among individual damage areas were statistically significant, a Bonferroni’s multiple comparison test was performed. Further, eye tracking metrics showed a statistically significant difference for all structural damage typologies like fixation duration, fixation count, visit duration, and visit count with adjusted *p* value ($\textit{p} < 0.05$). Table. 2 indicates the statistical analysis and their respective results for all damage regions considering fixation duration. For tabular results of the other metrics (fixation count, visit duration, and visit count) please refer to Supplementary Table. 1 for detail.

**Table 2:** Descriptive statistics of fixation duration for individual damage types showing their differences (see Supplementary Table 1, for detail on other metrics). For Building-1, there were no damage related to missing mortar and weathered stone 2 while crack and paint peeling off damage were not present for Building-2.

	Building-1			Building-2		
	Mean diff.	t-value	*p* value	Mean diff.	t-value	*p* value
*Fixation duration*						
Crack	1.179	16.07	<0.0001	–	–	–
Paint peeloff	− 0.1154	1.111	>0.99	–	–	–
Biological growth	0.8397	5.667	0.0053	1.025	6.147	0.0019
Surface stain 1	1.004	6.818	0.0017	0.6318	3.568	0.0512
Surface stain 2	0.9931	5.937	0.0024	1.410	9.664	0.0002
Missing mortar 1	–	–	–	1.461	10.47	0.0003
Missing mortar 2	–	–	–	1.669	11.98	0.0001
Weathered stone 1	0.7936	6.583	0.0007	1.243	11.48	<0.0001
Weathered stone 2	–	–	–	1.176	8.061	0.0001

## Discussion

This study examines human sense-making during human-structure interaction and how they assessed the building facade for damage. Based on the qualitative and statistical analysis for Buildings 1 and 2, it is evident that each participant has different way of looking at the structure and detecting damage on building facade. The overall strategy of the participants was to look at the structure holistically first and then scan specific parts of the structure element-wise. Participants with background knowledge of damage assessment and building diagnostics performed the element-wise inspection, while participants with less experience scanned the structure from top-to-bottom or right-to-left approach. In the end, they walk back from the structure and look back again to see if they missed any structural damage. There were some similarities and differences among participants that relate to their interest and prior knowledge in assessing the structure. Some participants (P6, P8) spent more time looking at the structure detecting damage, while others looked at the structure with lesser time, and this is clear from the result of Figs. 2 and 3 (see radar plot). This can also be evidenced by the fixation count and visit count with a higher number indicating more instances where they visit back-and-forth at certain damaged regions such as weathered stone, missing mortar, and surface stains. Based on statistical analysis, we find there was significant difference (*p* value of 0.05) among individual participants and how they assessed different damage regions. To further illustrate the fact that inspectors spent more time looking on damage regions versus the surrounding (non-damage) areas, Fig. 4 (area of interest) clearly shows the spatial distribution of all participants spending more time on damage areas and less time on surrounding (non-damage) areas of the structure.

Moreover, we also find how participant’s interest or prior knowledge can influence their decision-making. This study involves ten participants studying architectural engineering with good knowledge of structures and flow of forces etc. Based on the time spent on individual damage, we notice that crack damage was highly prioritized damage with highest fixation time followed by surface stains. This is true because the regions with these two damage can affect the structure more compared to other damage such as paint peeling off the window and or biological growth. This should be kept in mind that all participants performed the experiments separately so the study was not compromised or influenced by other participant’s behavior or actions. From a statistical perspective, the results also indicate the difference between all the individual damage regions and the surrounding except paint peeling off region which was found to not be significantly different from the surrounding areas. Further, it must be noted that not all the damage were inspected and assessed by all the participant’s given participants backgrounds and knowledge. It is possible that some damage were missed during structural assessment (see Supplementary Figs. 5–44 V for detail) according to their individual heat maps and gaze plots. In the future study, we will incorporate the damage type that were missed and assign a metric that indicates how crucial a damage type is from the structures perspective. This research has potential implications for training data as we move from expert knowledge capture towards path planning with unmanned systems– UAVs or UGVs. Understanding these complex human-building interactions will inform future robotic replication of an inspectors reasoning and decision-making abilities.

It can be argued that the results may have some bias due to the lighting condition depending on the time of the day, nearby people walking randomly distracting them during an inspection, and their prior experience. This was significantly seen for Participants 1, 3, and 4. In addition, factors such as the subject and environmental condition also bias the inspector’s assessment for façade inspection. Based on the above discussion, it can be concluded that a participant’s inspection can be biased depending on their prior experience and knowledge about structure assessment and façade inspection. Someone with prior work with damage detection and structure assessment would identify or inspect structures differently than someone without experience. Similarly, participants’ interest in whether the structure is a heritage building or a historic structure would also make a difference while looking at structure elements for details. Moreover, these assessments can come at the cost of inspection time and cognitive stress and influence their decision-making and overall reasoning. However, taking into account these biases was not part of this study and will be explored as a future case study.

## Conclusions

The ability of an autonomous system to understand human intent and implicit attention is essential for many decision-making and reasoning tasks. In this work, eye tracking was used to capture the inspectors expert knowledge about a structure and how they perform an overall assessment of the facade structure. This study analyzed eye tracking metrics based on fixations and visits to understand different inspectors gaze patterns and how they looked at a structure during an inspection. We experimented on two different facades to assess any differences or similarities in the pattern of general observation to detect damage. The study was conducted with 10 participants with fair distribution of gender and experience. The two building facades had different damage typologies, and they were situated in locations at the University Park campus.

The most important metrics for analyzing eye movements are fixations and visits and how they correlate with their saccadic actions. Metrics based on fixation, such as fixation duration and fixation count, and visit, such as visit duration and visit count, were discussed and analyzed in detail for both case studies. It is interesting to note that longer fixation duration can both relate to a thorough and detailed inspection, and at the same time, it can imply that participants were suffering from lack of attention. The implications of this for training UAVs and UGVs needs to be further studied in the future research works. Moreover, we learned of several possible diagnostic biases that can affect the performance of building inspection, which were significant head and body motion, eye tracker’s calibration, and distance to the structure. For this case study, we have noticed a bias in the structure assessment due to the tall building facade, and participants were more focused on the lower facade portion to detect damage. Again, the implications for the effect of this on robotic training data needs to be further studied. In conclusion, fixation and visit metrics aggregated with gaze trail can inform participants’ behavior and interest points. Eye tracking can be helpful in training inspectors with no prior knowledge or less experience in damage assessment to perform damage diagnosis efficiently and on time.

Future studies will extend this work to include field inspectors and practitioners to capture their expert knowledge for building assessment and damage diagnosis. Further, we will use the preliminary data from this study to develop a path planning technique for fast and efficient damage localization for unmanned systems. To integrate this work with state-of-the-art drone based inspection, we will make a saliency metric and quality metric which will be used for understanding what type of damage is important and crucial from structural point of view and the collected data is good enough to be relied upon for future assessments. Further, the attention mechanism will also help inform better decision making when paired with eye movements data.

## Supplementary Information

Supplementary Information.

## Data Availability

The data that support the findings of this study are openly available in Zenodo https://doi.org/10.5281/zenodo.7125956.
