# Supplementary material for: Analysis of gaze patterns during facade inspection to understand inspector sense-making processes
Source: Sci Rep. 2023 Feb 20;13:2929. doi: 10.1038/s41598-023-29950-w (PMC9941087; doi:10.1038/s41598-023-29950-w)
Supplement: Supplementary file 1 — Supplementary Information. [file 41598_2023_29950_MOESM1_ESM.pdf]

Appendix I

Experimental design prompt

Participants were given an experimental prompt before the start of the experiment and were asked to perform the task of structural assessment accordingly. The study followed the ethical guidelines of Human Subjects Research and was approved by the Institutional Review Board (IRB) for the Human Research Protection Program (HRPP) of The Pennsylvania State University (STUDY00016625).

You are invited to participate in a research study. Research studies include only people who voluntarily choose to take part. This summary explains critical information about this research. You are urged to ask questions about anything unclear to you.

- *Consider the section of the building that you can see from here and assess the condition of the building section.*
- *Please describe out loud any damage or peculiarities that you see.*
- *You must pretend I am not here, so I cannot answer any questions once you have started the experiment.*
- *You are allowed to move your head and body as well as walk if you would like.*
- *You may take however long you wish to do the task.*
- *We are not evaluating your abilities, and no information from this volunteer work will impact you.*

If you agree and would like to participate in the survey, please continue to the next page.

Appendix II

Visualization of Gaze plot and Heat map

The heat map describes the overall distribution of a participant's vision over a particular stimulus. The areas with red color indicate the high intensity of a participant's fixations compared to low-intensity areas indicated by dark blue. Heat maps can effectively document visual attention in a scene and consider all the fixations of a participant; this can be used to understand decision-making processes. Similarly, individual gaze plots vary based on a specific person's fixations and scanning strategies which correspond to their gaze trail and involuntary, saccadic eye movement. The longer the fixation duration, the larger the circle diameter; the number inside the circle indicates the order of where the participants looked.

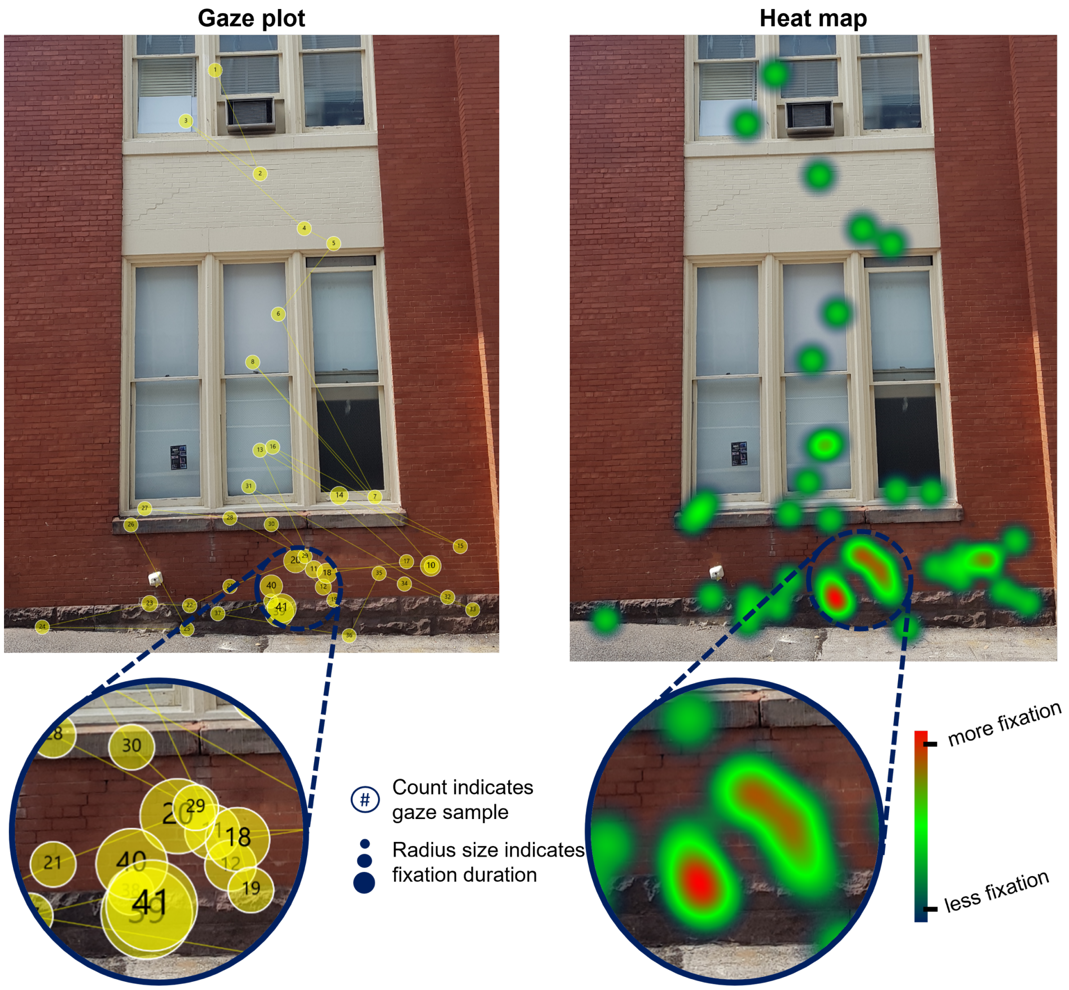

Supplementary Figure 1. Visualization of eye tracking metrics: (left) Gaze plot showing participant fixation, the line indicates trail between two successive fixations, radius indicates duration, and count indication number of fixation samples in time; (right) Heat map showing the concentration of participant gaze in time.

Appendix III

Data filtering (raw vs. fixation vs. attention)

The velocity-threshold identification (I-VT) filter is a velocity based classification algorithm and is used to classify between fixations and saccades. We have shown a comparison of different filtering techniques below for one of the data collected for our designed experiment. Raw filter (see Fig. 1) is the one with no data cleaning and processing, and the gaze plot shows the participant’s fixations every msec. Fixation filter (see Fig. 2) indicates participant’s fixations where we set up the velocity threshold to be 30°/sec by default and noise reduction is applied by using moving median filter. Fixations that do not falls under this threshold bound are removed. Attention filter (see Fig. 3) indicates fixations with slightly more directional shifts of the eye and the threshold for this filter is 100°/sec by default. It is important to note that the threshold is increased for attention filter, its because this filter is used for data collected from wearable eye tracker (outdoor with more dynamicity) with more head and body movement comparing to fixation filter where the data is collected from screen based eye tracker (indoor with more stability) with less head and body movement.

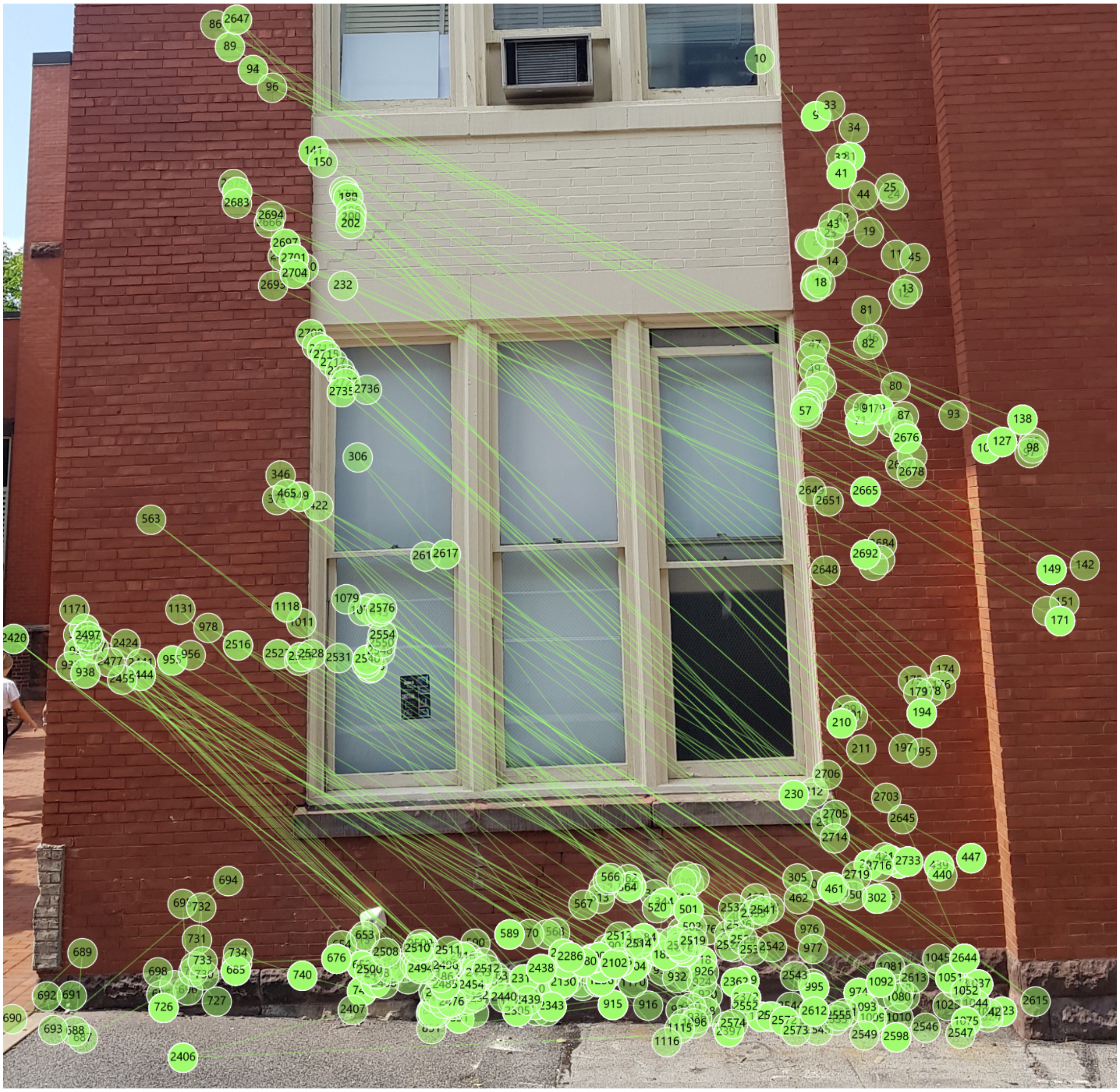

Supplementary Figure 2. Gaze plot of participant using raw filter settings

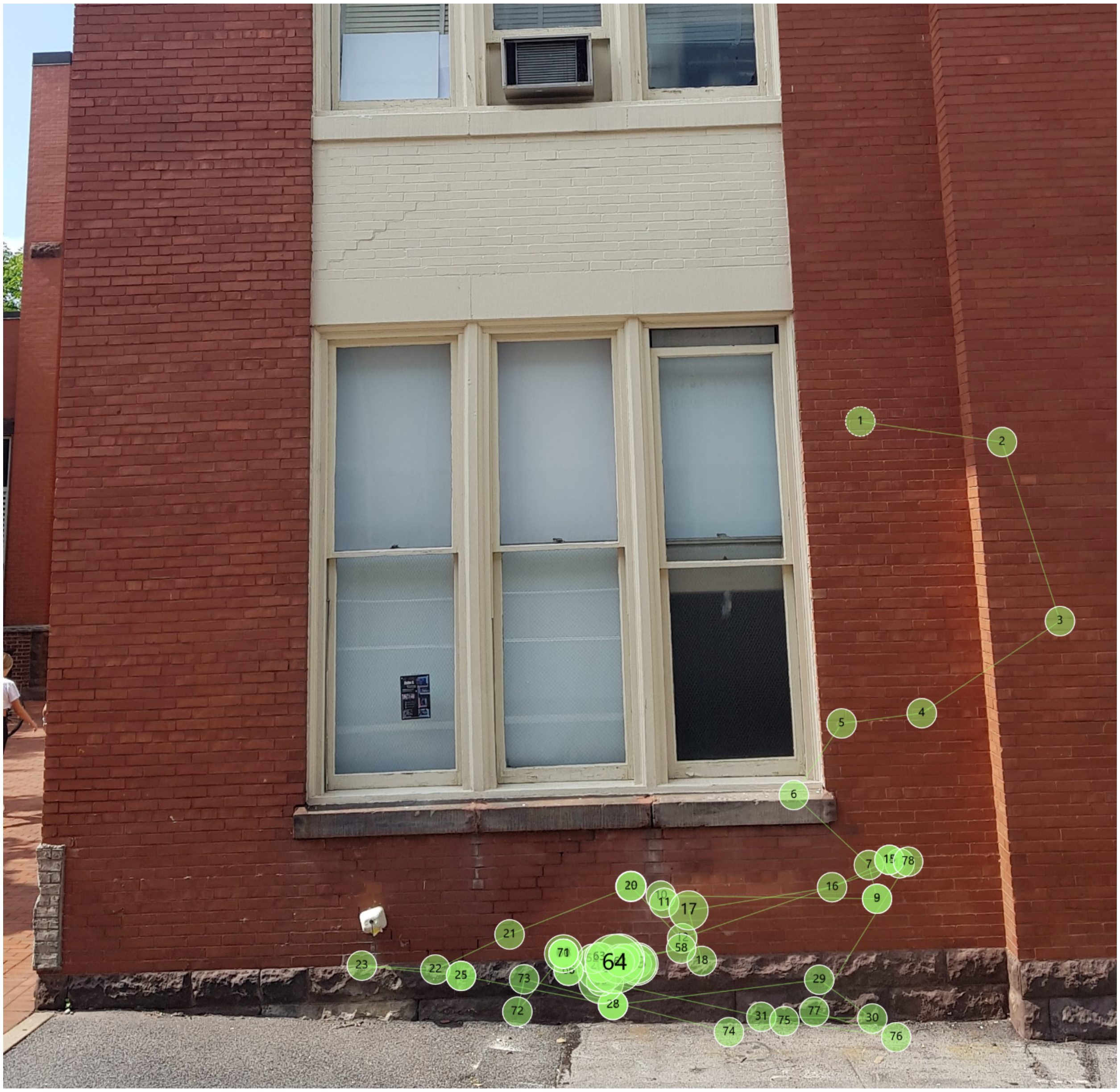

Supplementary Figure 3. Gaze plot of participant using fixation filter settings

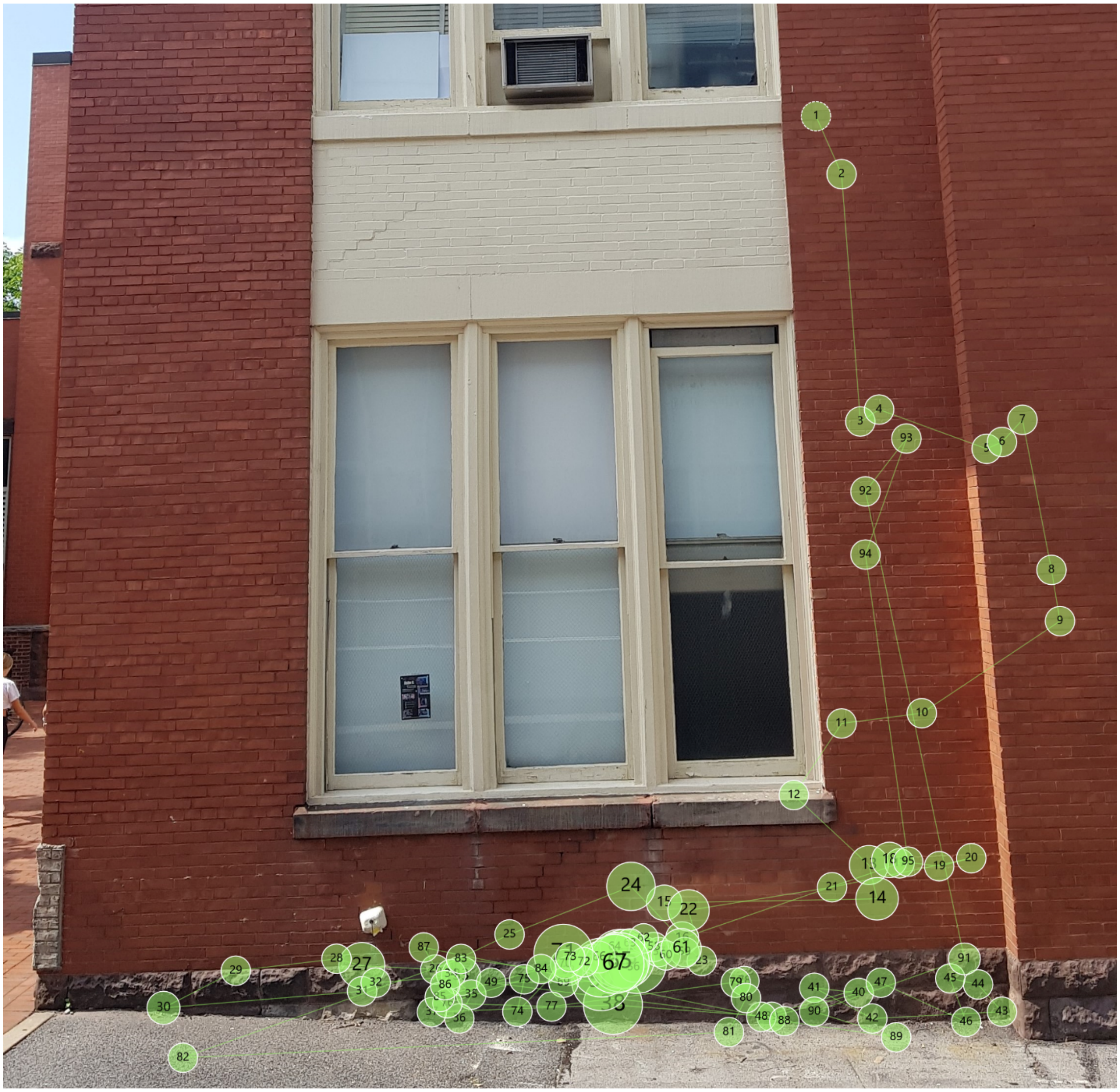

Supplementary Figure 4. Gaze plot of participant using attention filter settings

Appendix IV

Eye tracking strategy coupled with audio data for preliminary bias assessment

All participants have different scanning strategies and methodologies for assessing the structure. Participants with a background in historic structure and damage assessment looked at masonry bricks, cracks, and foundation damage compared to other participants who looked at surface staining, biological growth, and paint peeling damage. The correlation among participants was seen since the complete structure was scanned, and the difference was among the time spent versus back-and-forth visits among damage types. Figs. 3 and 4 indicate the heat map and gaze plot showing similarities, while statistical analysis was made to perform correlation and prove that there is a significant difference among participants for looking at different damage types.

In addition to eye movements data, audio data was collected and analyzed during pre-processing stage using Glasses 3. The gaze samples that were false positive were corrected manually using the raw data samples. The missed gaze data was placed according to the participants' descriptions of what they were looking at. The observer took these notes in the form of comments and audio notes. Gaze samples and audio and video data were mapped together on an image for further qualitative and statistical analysis. After post-mapping, the observer reviewed the data again to check for any incorrect or missed gaze data that would compromise the findings. In the end, statistical analysis was performed on the corrected data.

Appendix V

Heat maps and Gaze plots (Building-1)

Participant-1

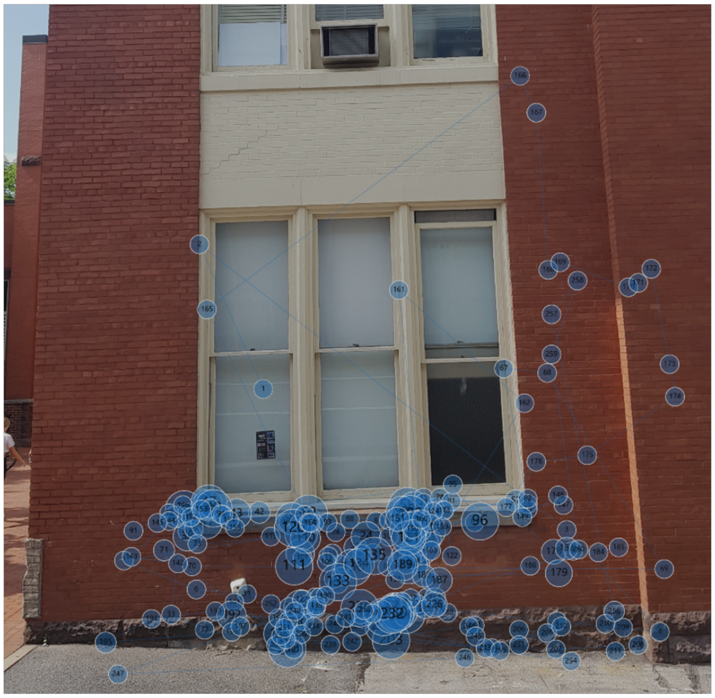

Supplementary Figure 5. Gaze plot of participant 1

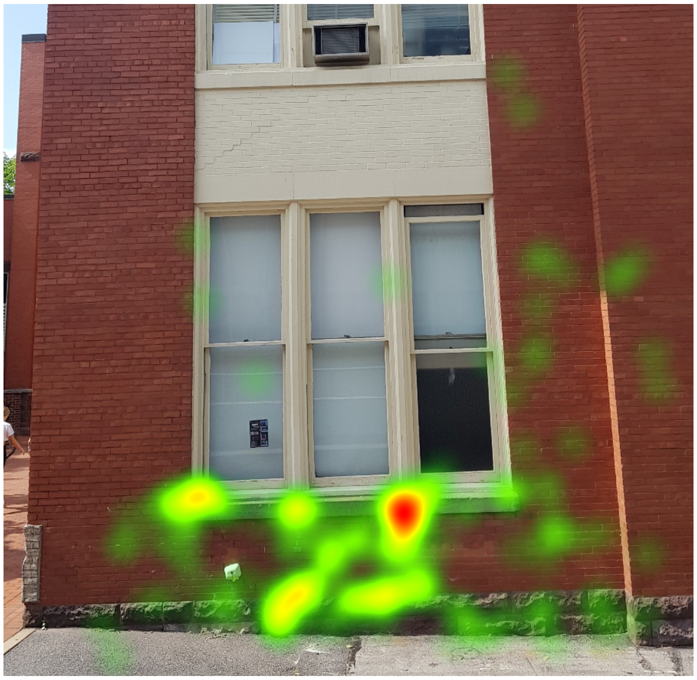

Supplementary Figure 6. Heat map of participant 1

Participant-2

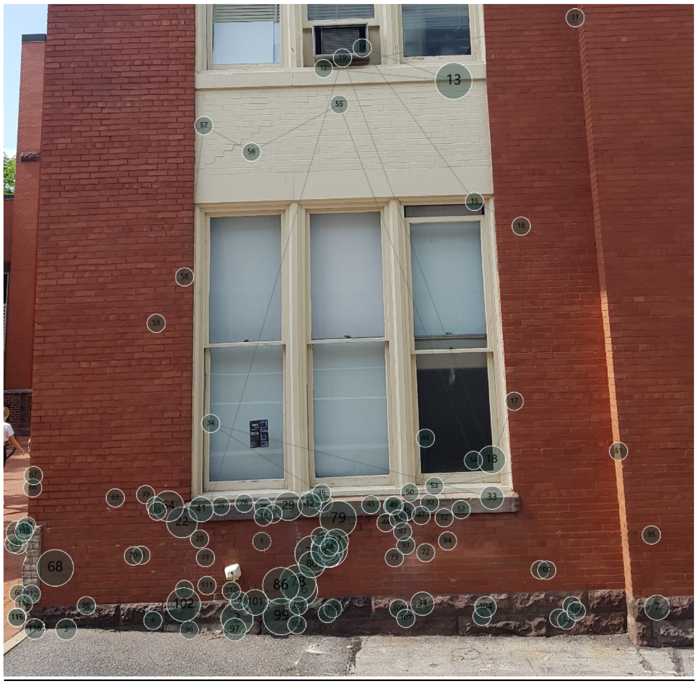

Supplementary Figure 7. Gaze plot of participant 2

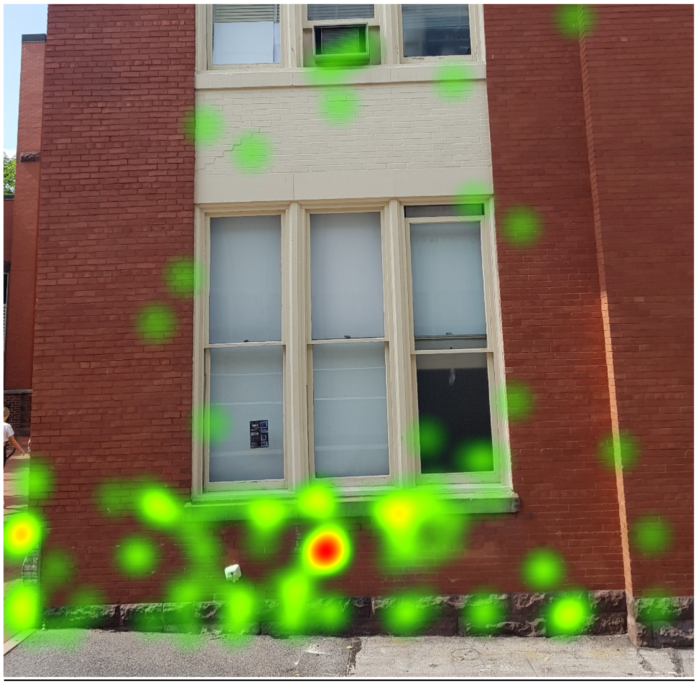

Supplementary Figure 8. Heat map of participant 2

Participant-3

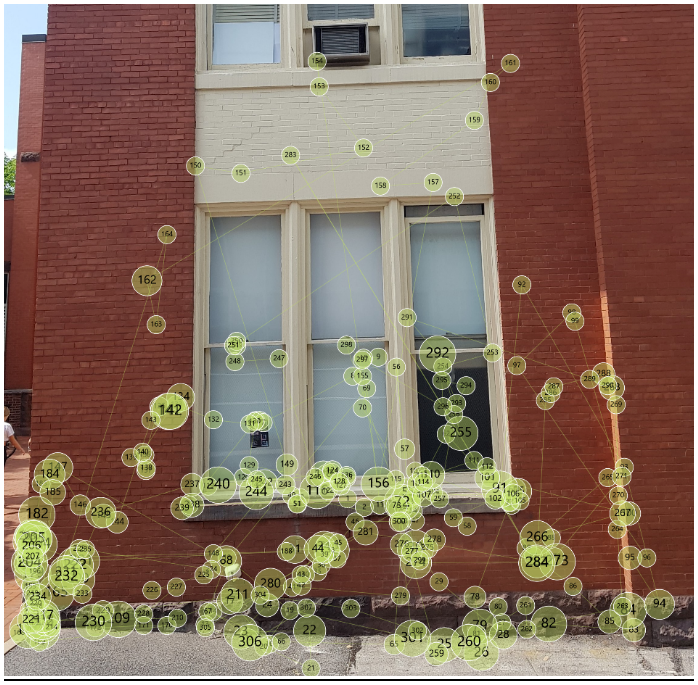

Supplementary Figure 9. Gaze plot of participant 3

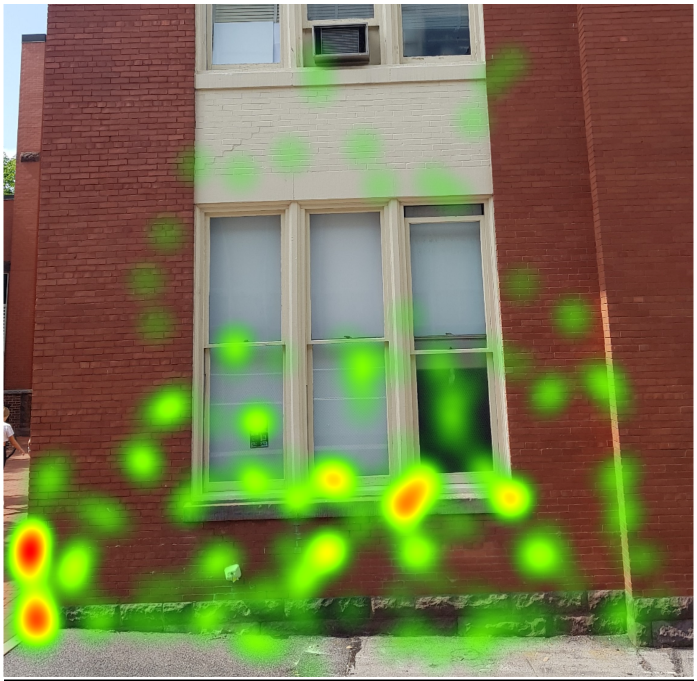

Supplementary Figure 10. Heat map of participant 3

Participant-4

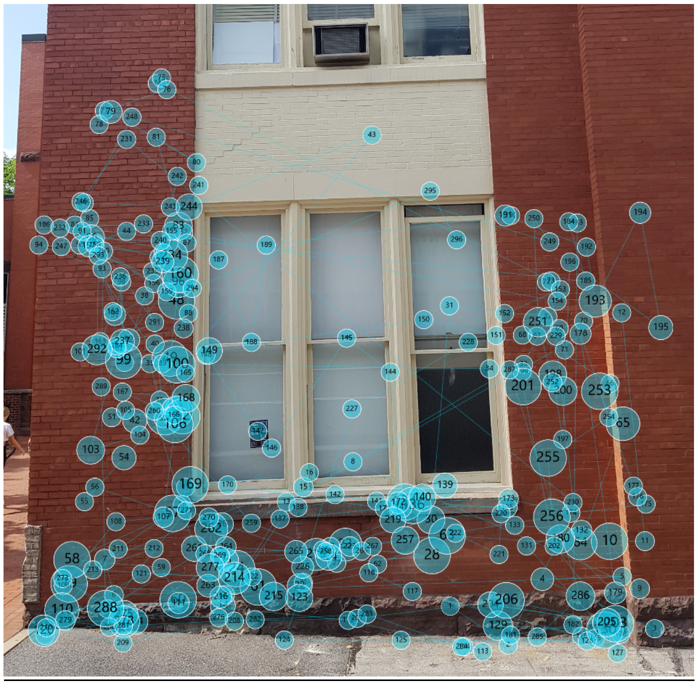

Supplementary Figure 11. Gaze plot of participant 4

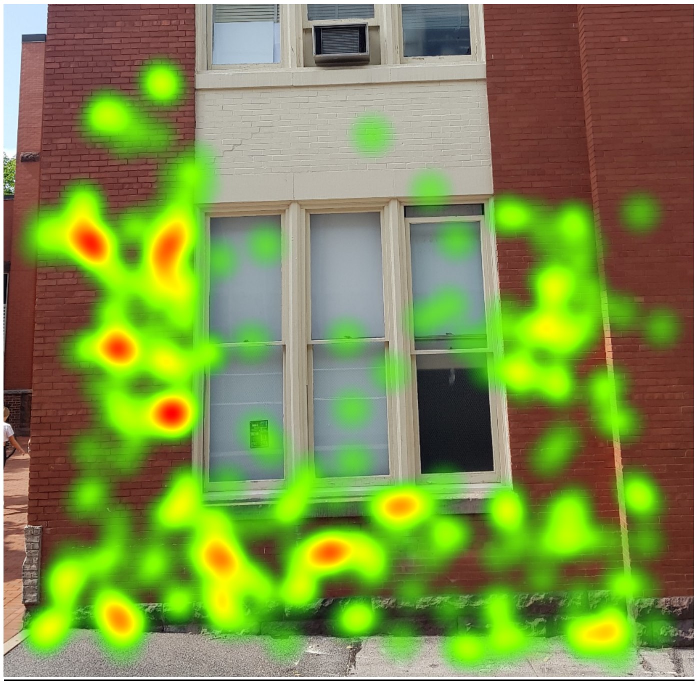

Supplementary Figure 12. Heat map of participant 4

Participant-5

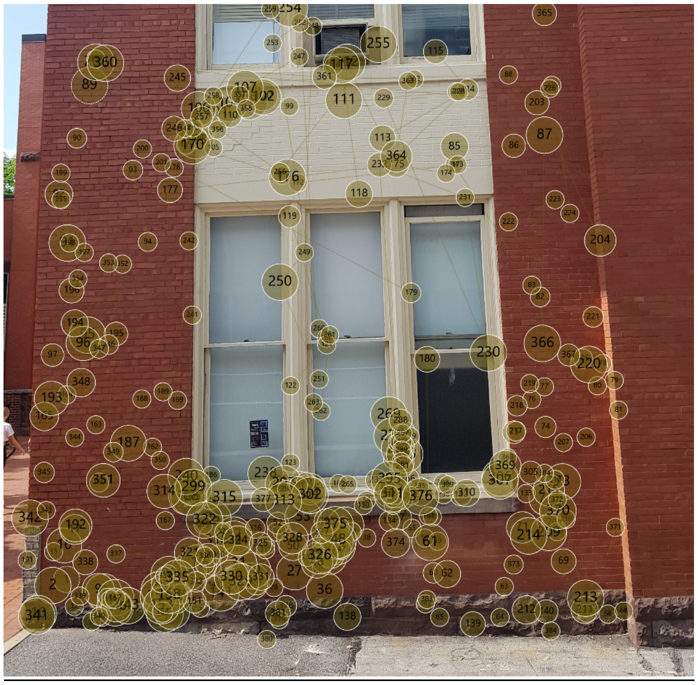

Supplementary Figure 13. Gaze plot of participant 5

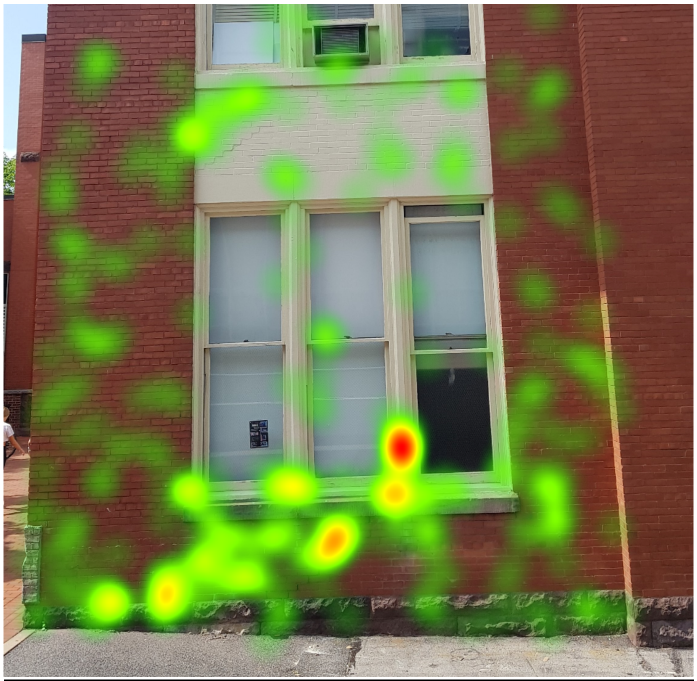

Supplementary Figure 14. Heat map of participant 5

Participant-6

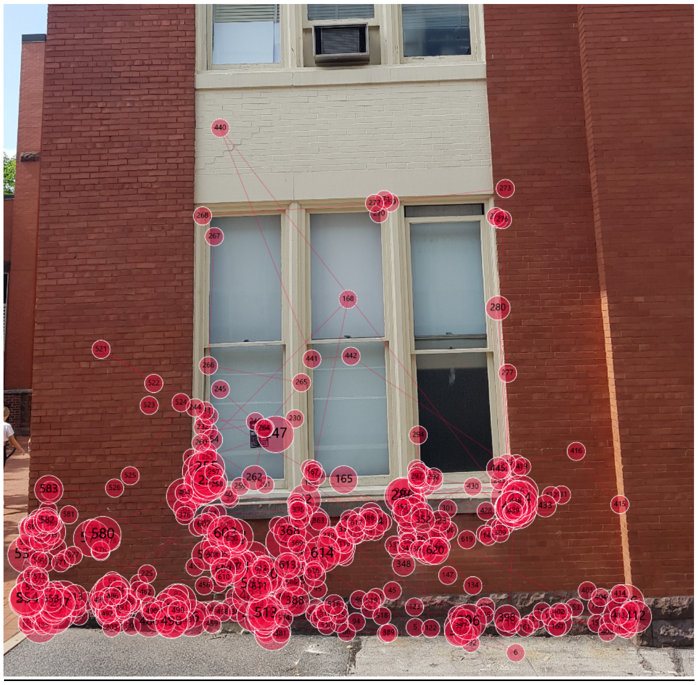

Supplementary Figure 15. Gaze plot of participant 6

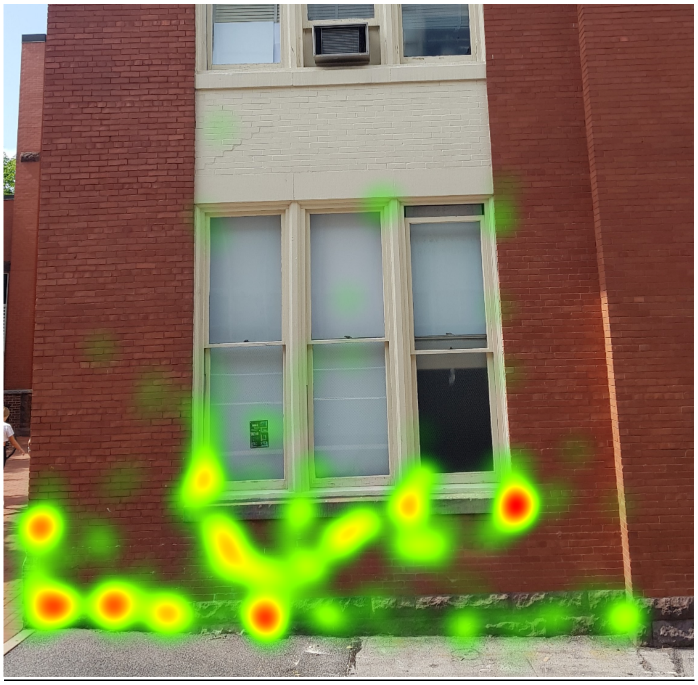

Supplementary Figure 16. Heat map of participant 6

Participant-7

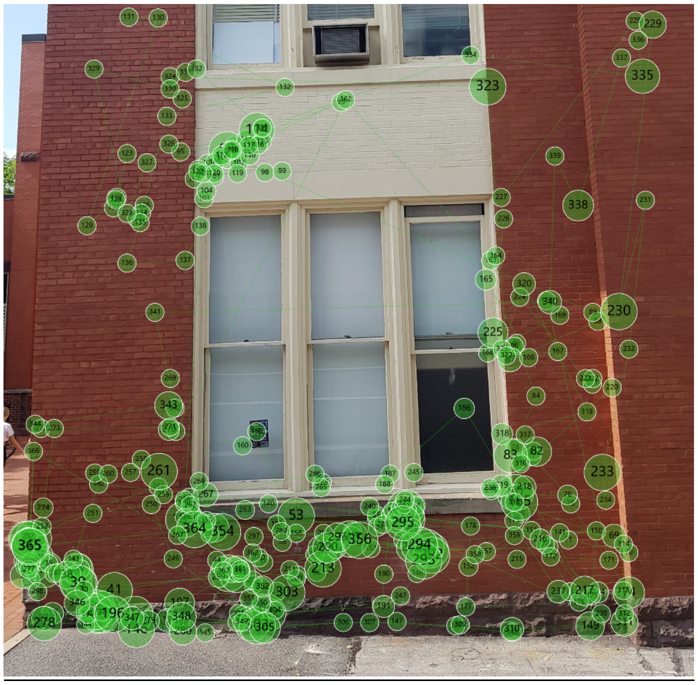

Supplementary Figure 17. Gaze plot of participant 7

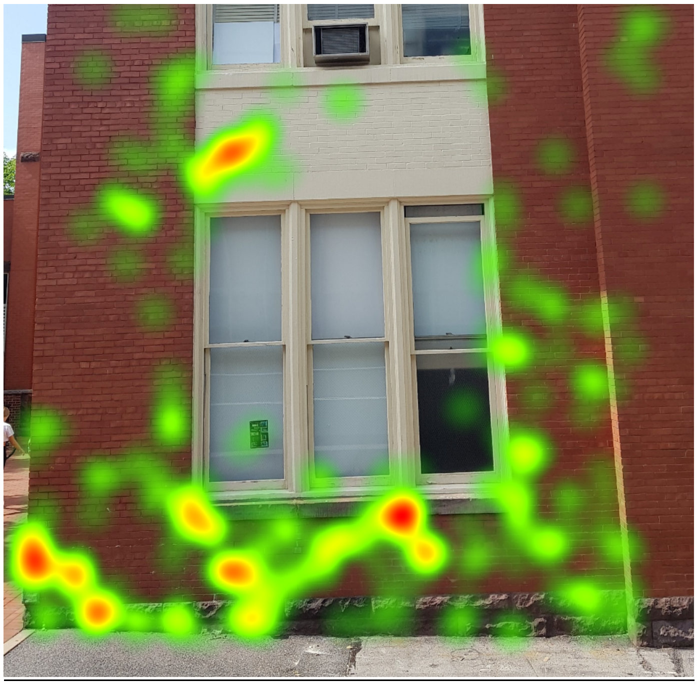

Supplementary Figure 18. Heat map of participant 7

Participant-8

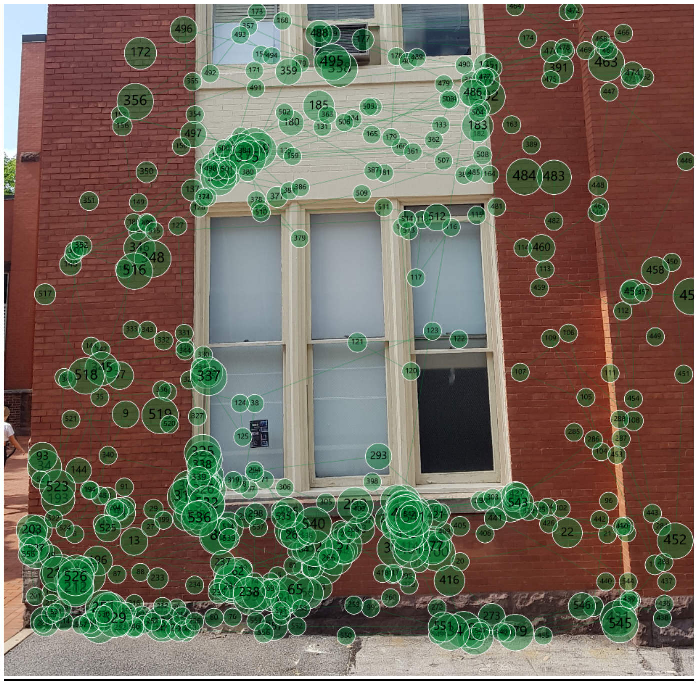

Supplementary Figure 19. Gaze plot of participant 8

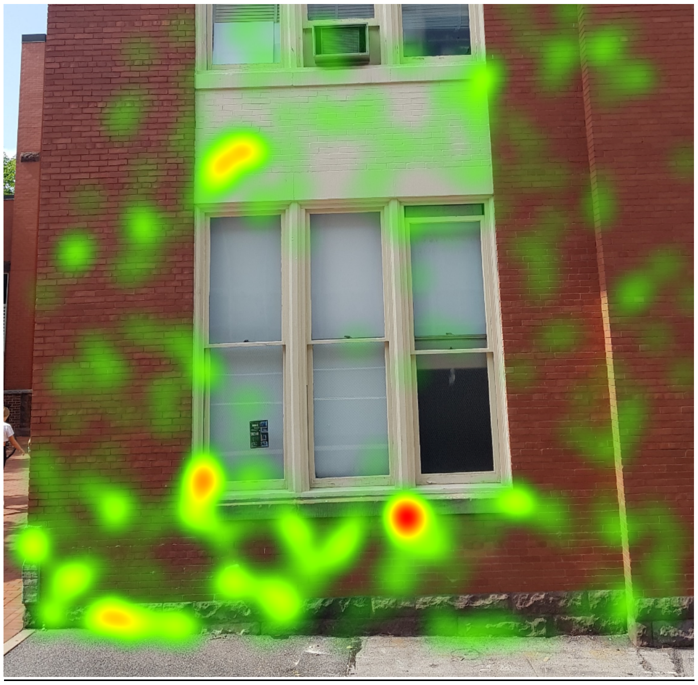

Supplementary Figure 20. Heat map of participant 8

Participant-9

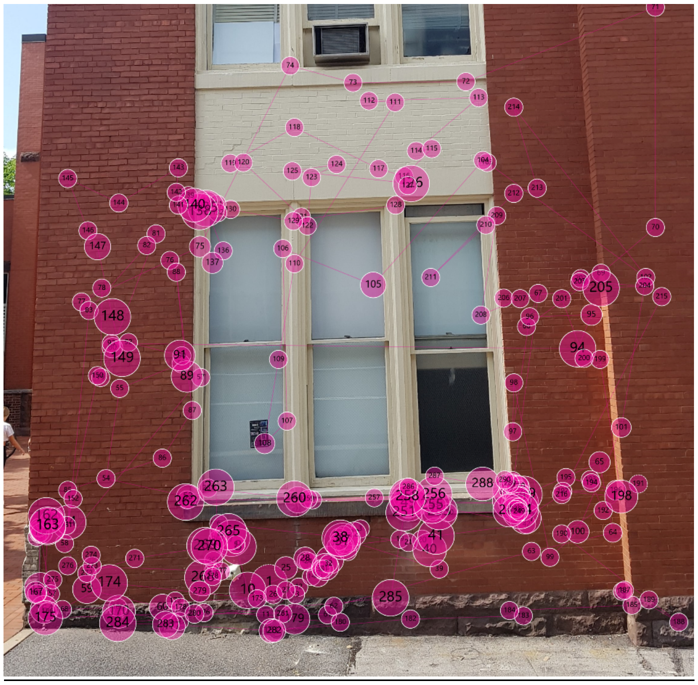

Supplementary Figure 21. Gaze plot of participant 9

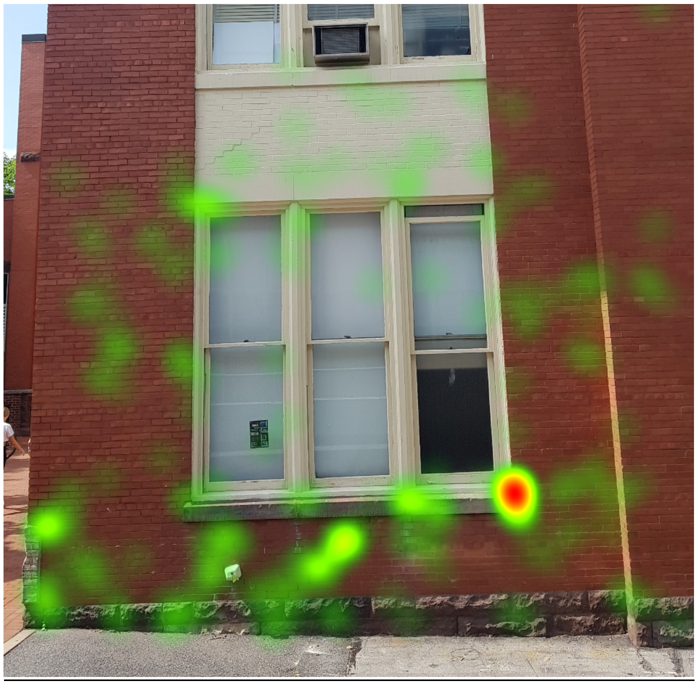

Supplementary Figure 22. Heat map of participant 9

Participant-10

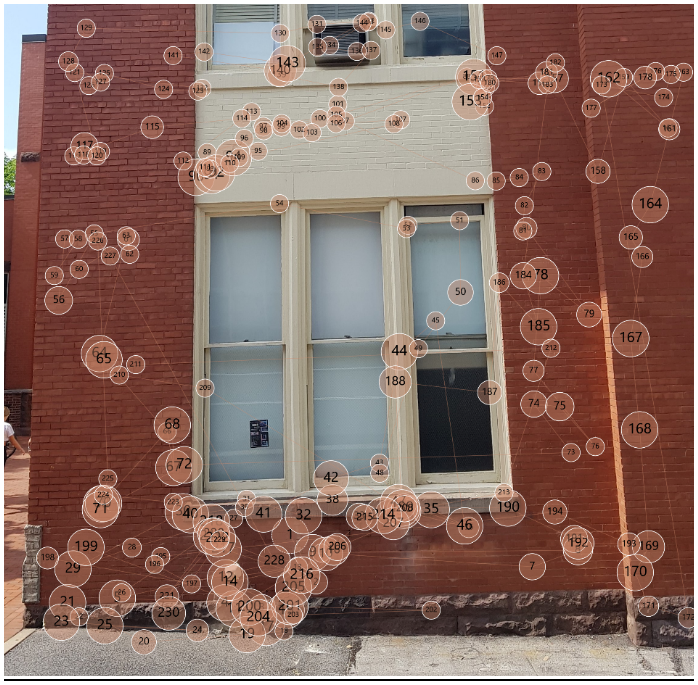

Supplementary Figure 23. Gaze plot of participant 10

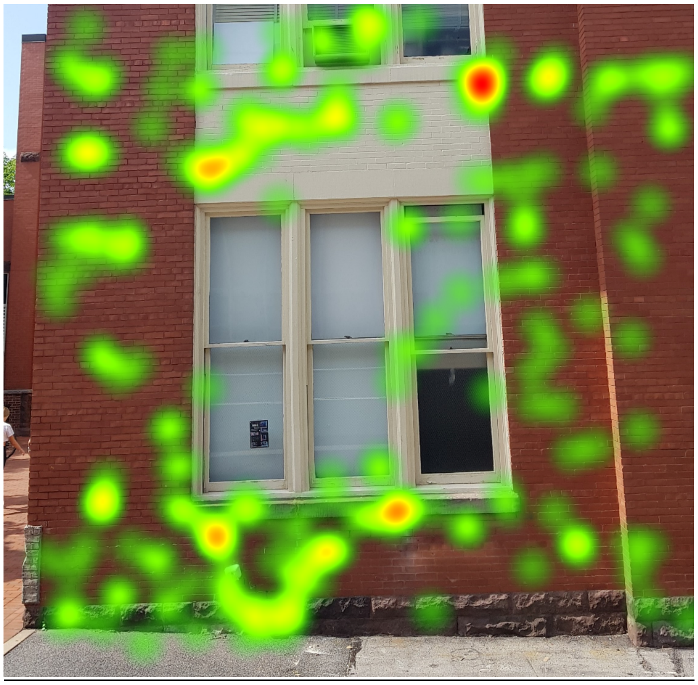

Supplementary Figure 24. Heat map of participant 10

Heat maps and Gaze plots (Building-2)

Participant-1

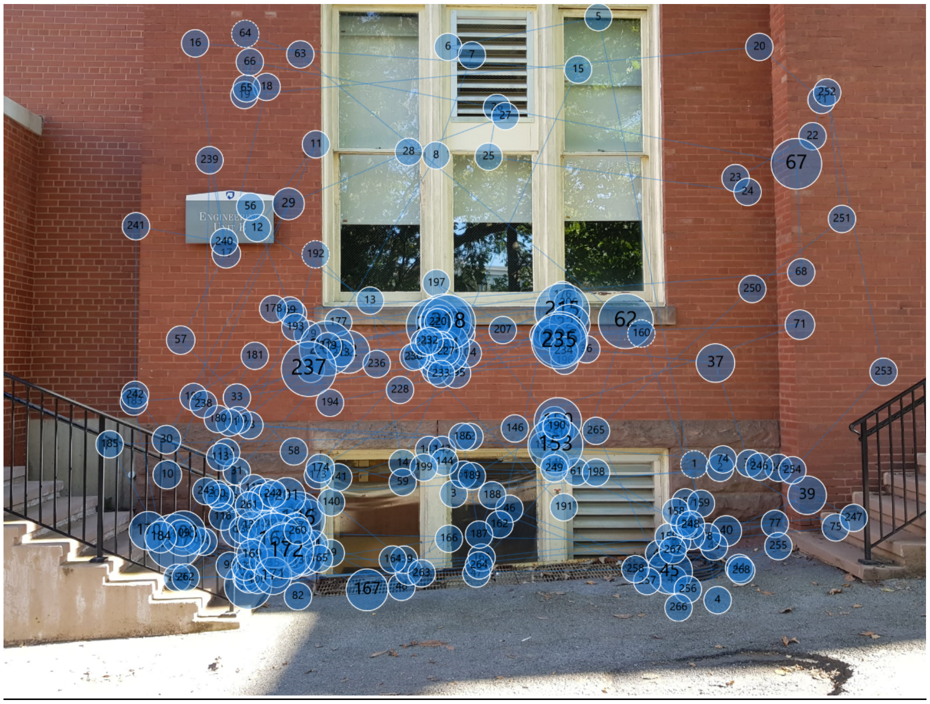

Supplementary Figure 25. Gaze plot of participant 1

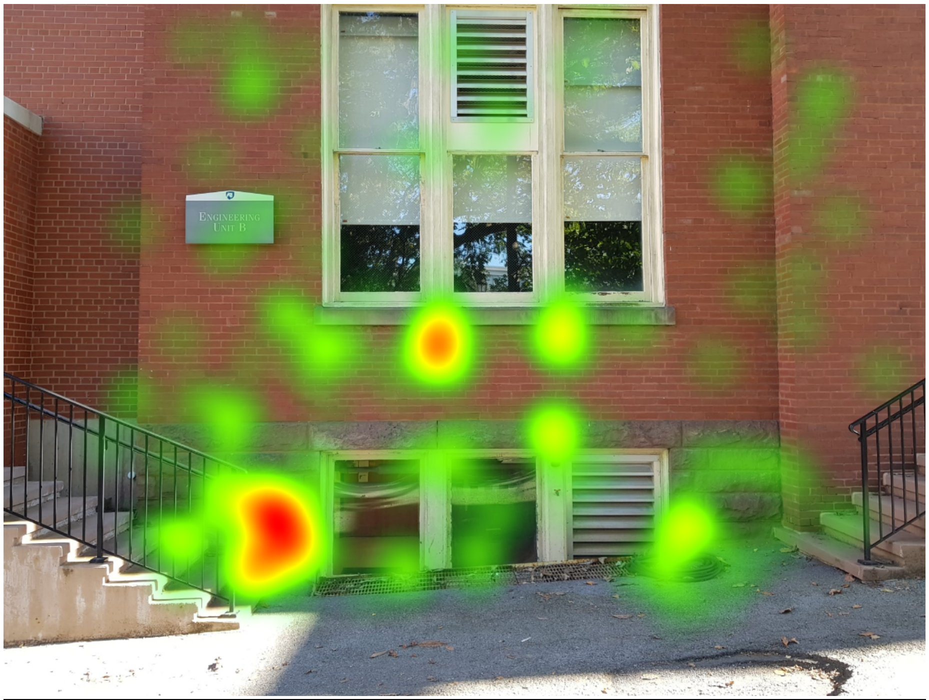

Supplementary Figure 26. Heat map of participant 1

Participant-2

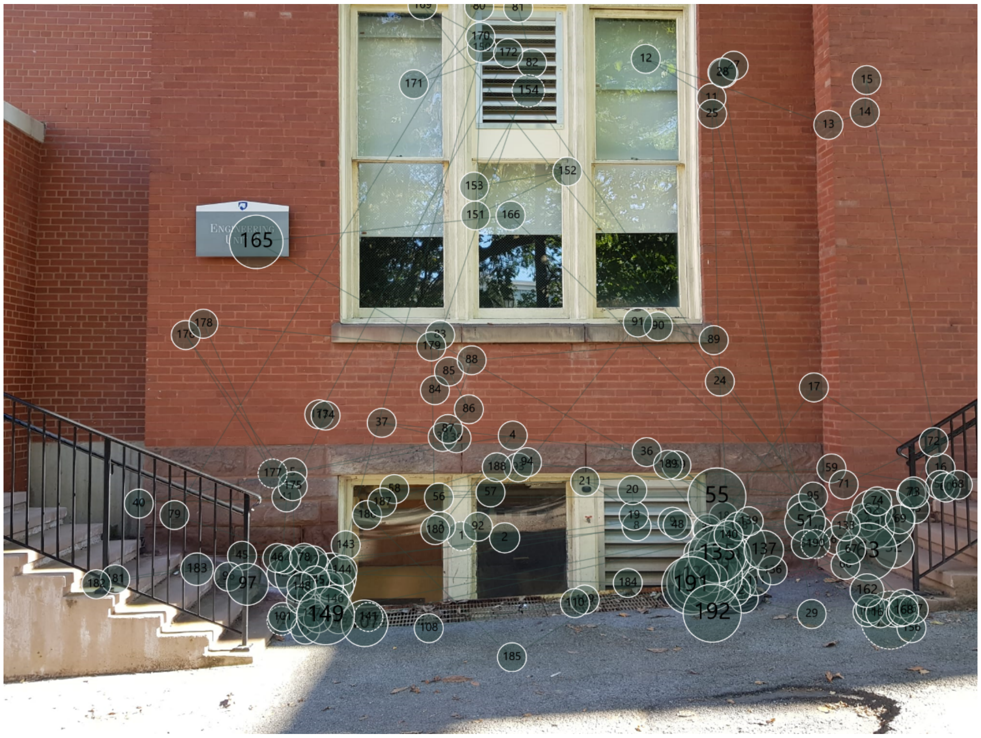

Supplementary Figure 27. Gaze plot of participant 2

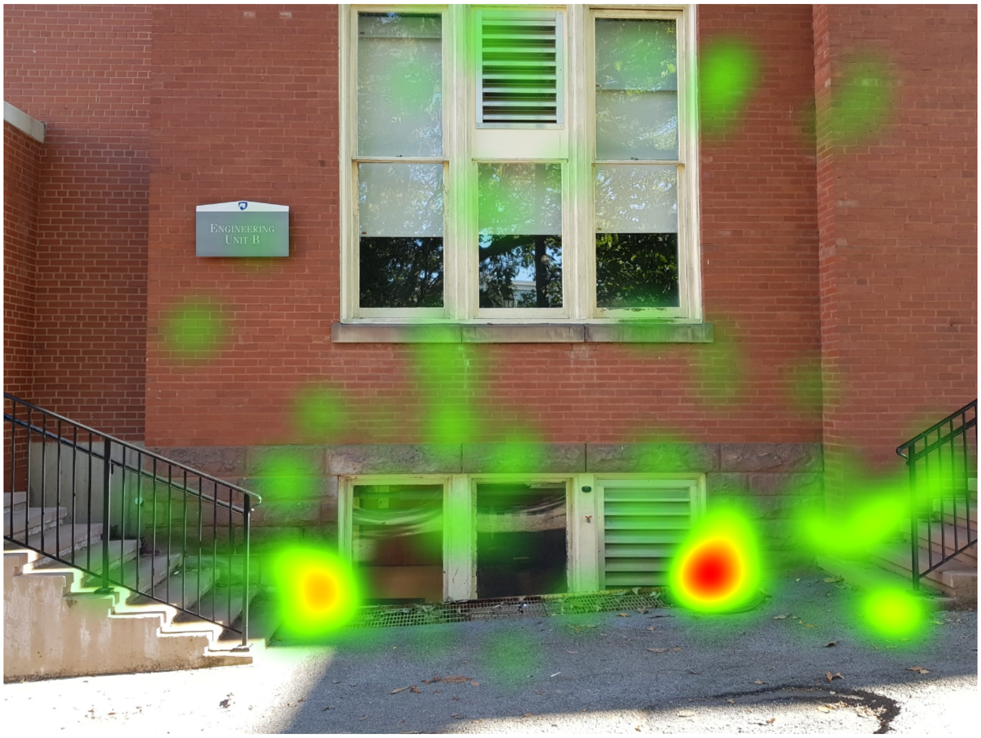

Supplementary Figure 28. Heat map of participant 2

Participant-3

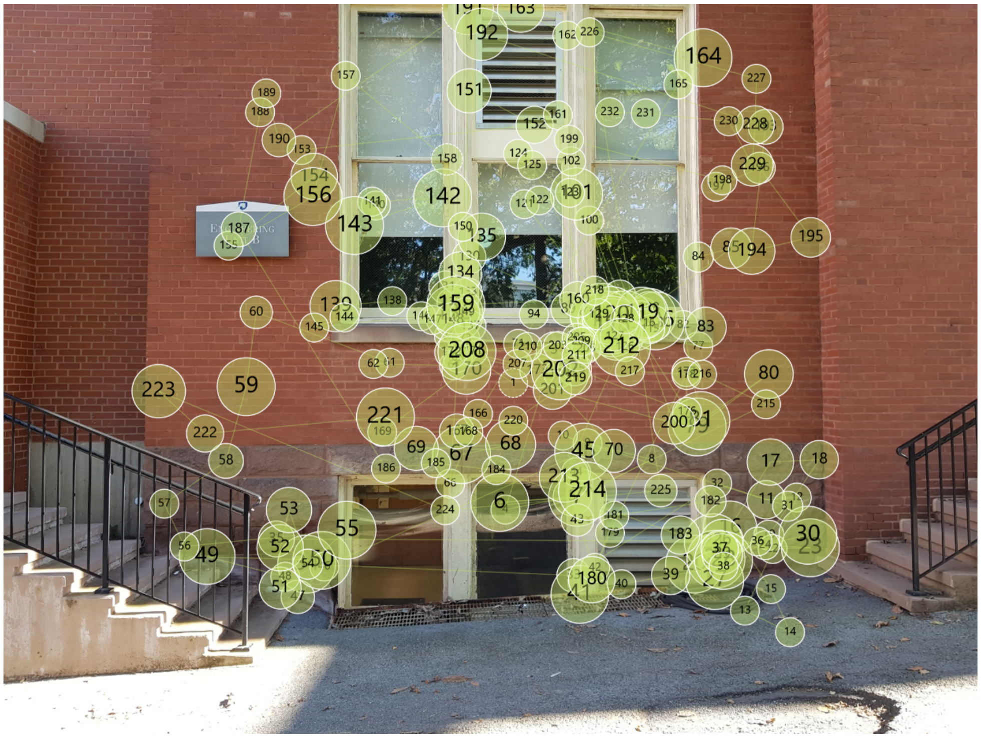

Supplementary Figure 29. Gaze plot of participant 3

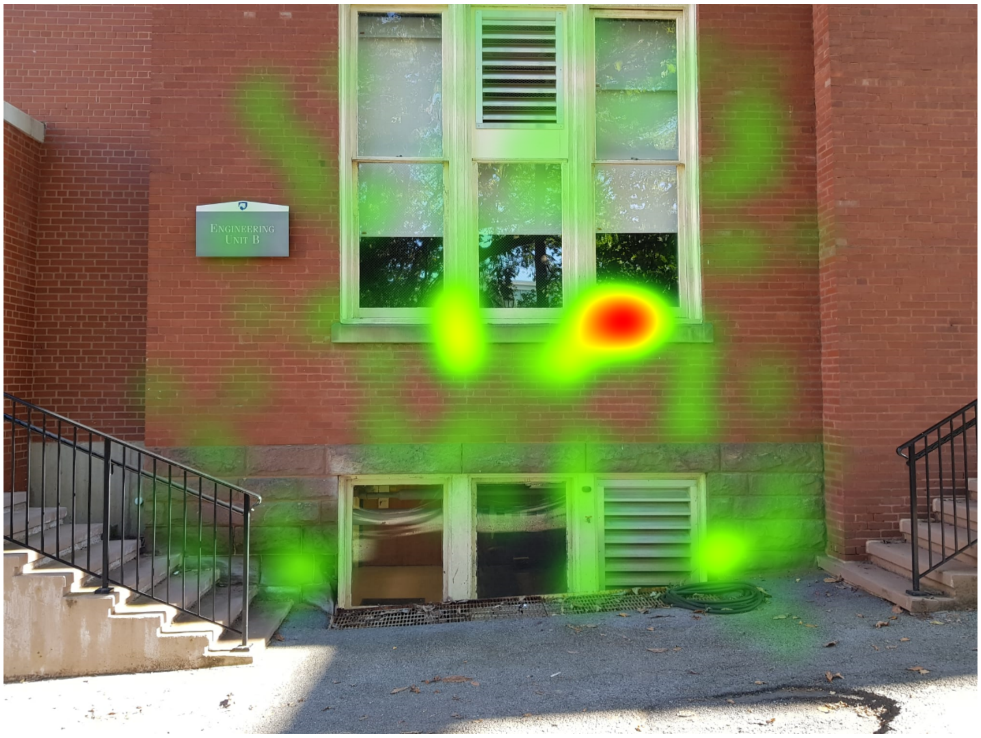

Supplementary Figure 30. Heat map of participant 3

Participant-4

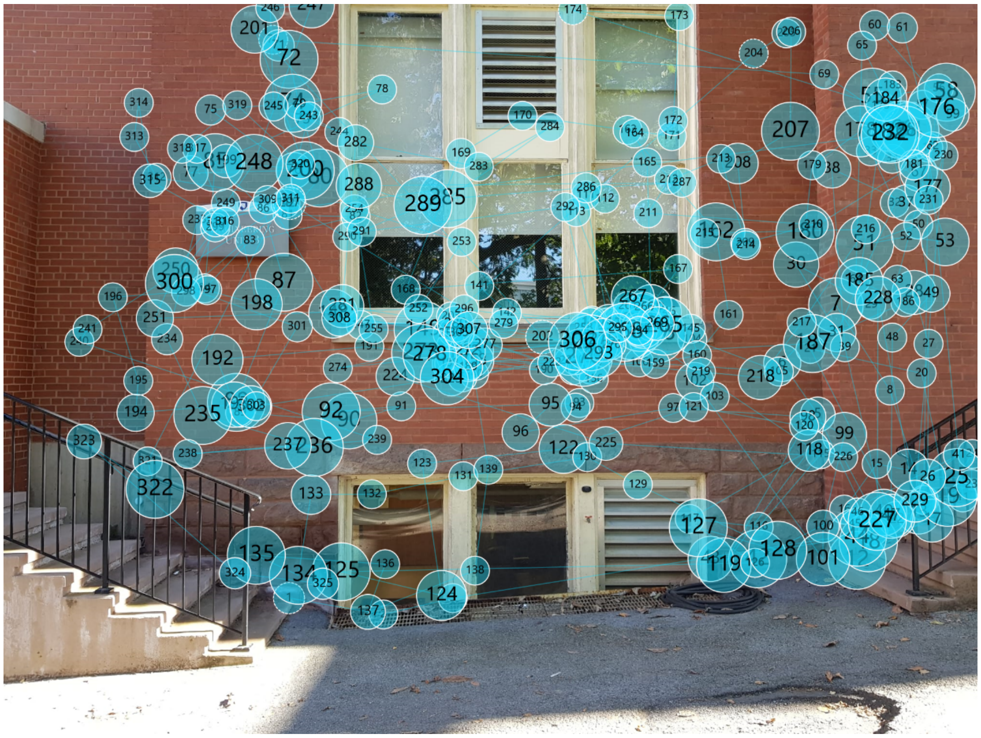

Supplementary Figure 31. Gaze plot of participant 4

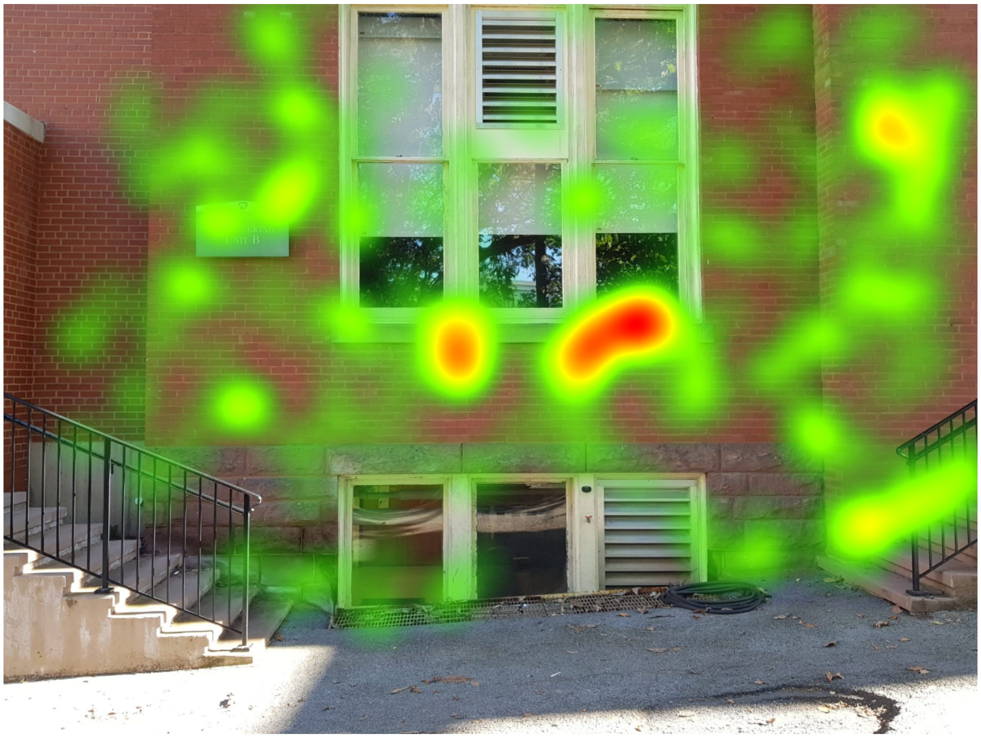

Supplementary Figure 32. Heat map of participant 4

Participant-5

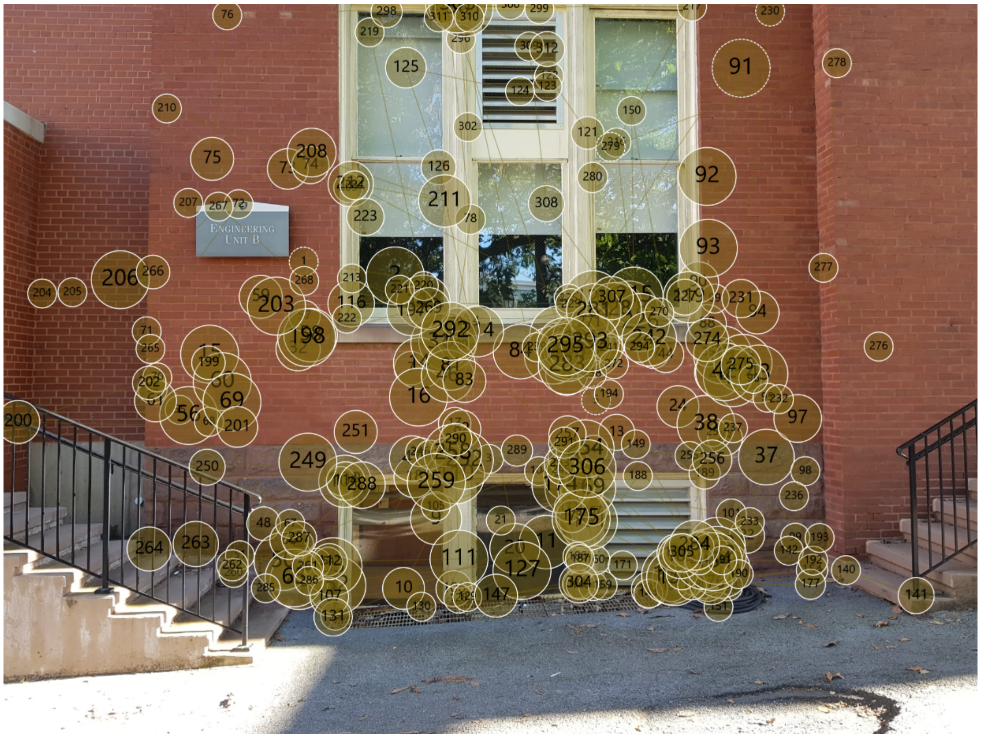

Supplementary Figure 33. Gaze plot of participant 5

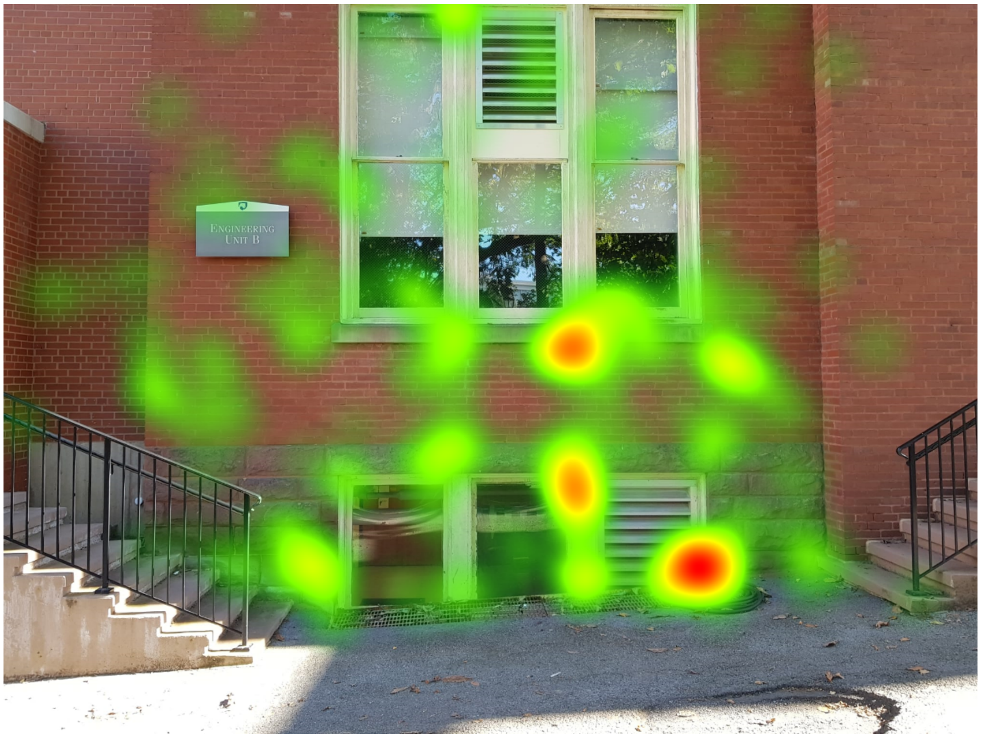

Supplementary Figure 34. Heat map of participant 5

Participant-6

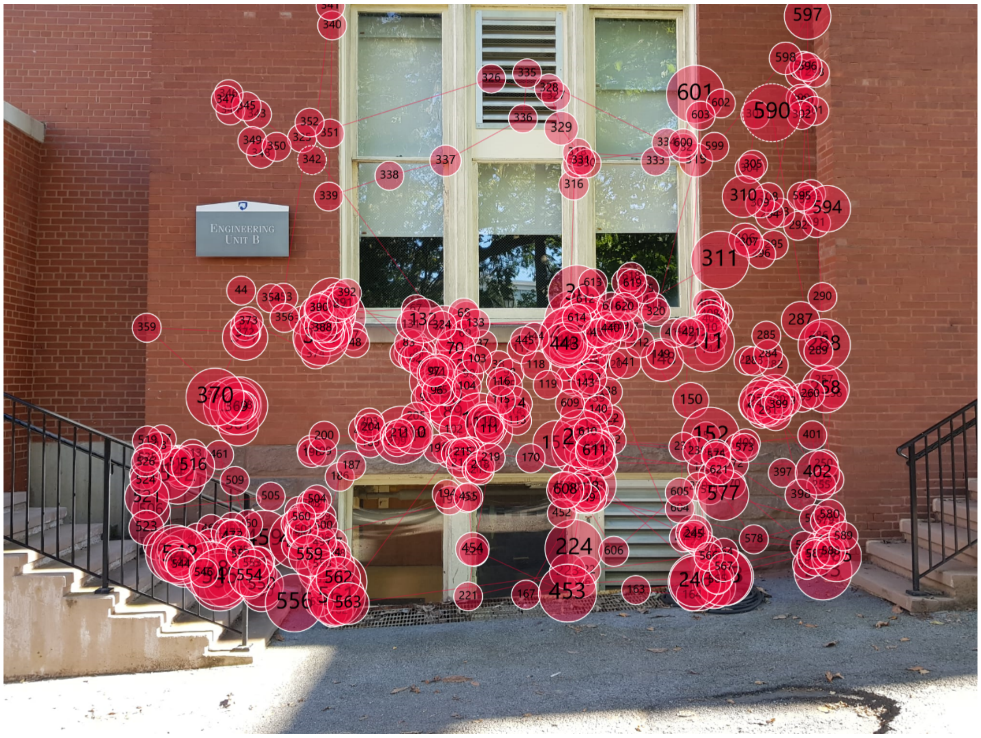

Supplementary Figure 35. Gaze plot of participant 6

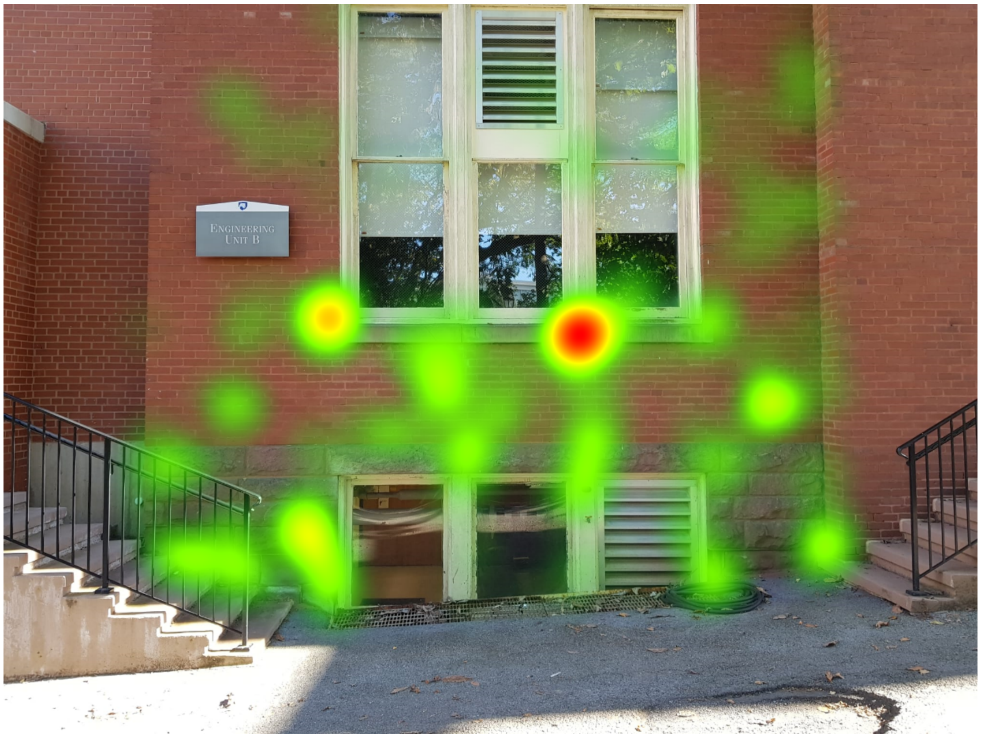

Supplementary Figure 36. Heat map of participant 6

Participant-7

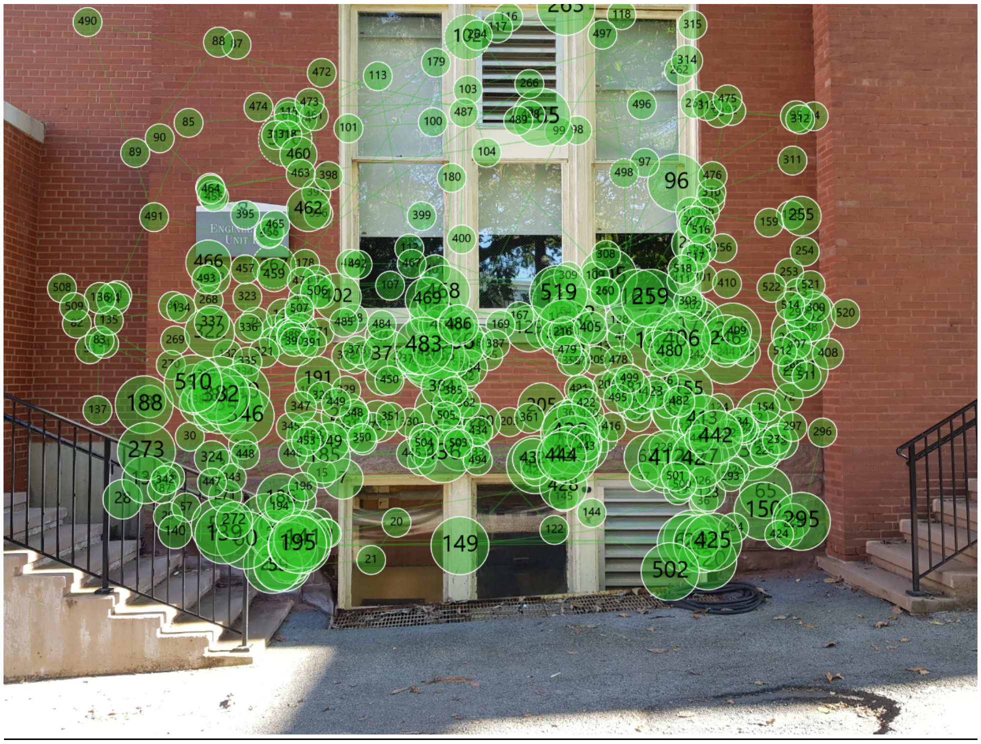

Supplementary Figure 37. Gaze plot of participant 7

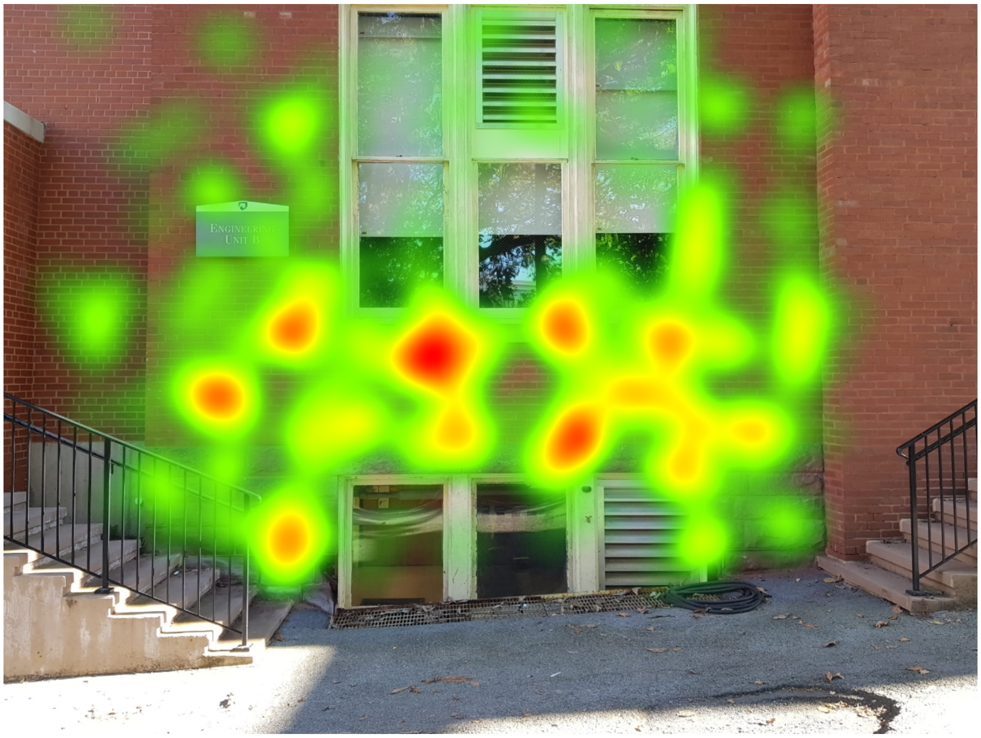

Supplementary Figure 38. Heat map of participant 7

Participant-8

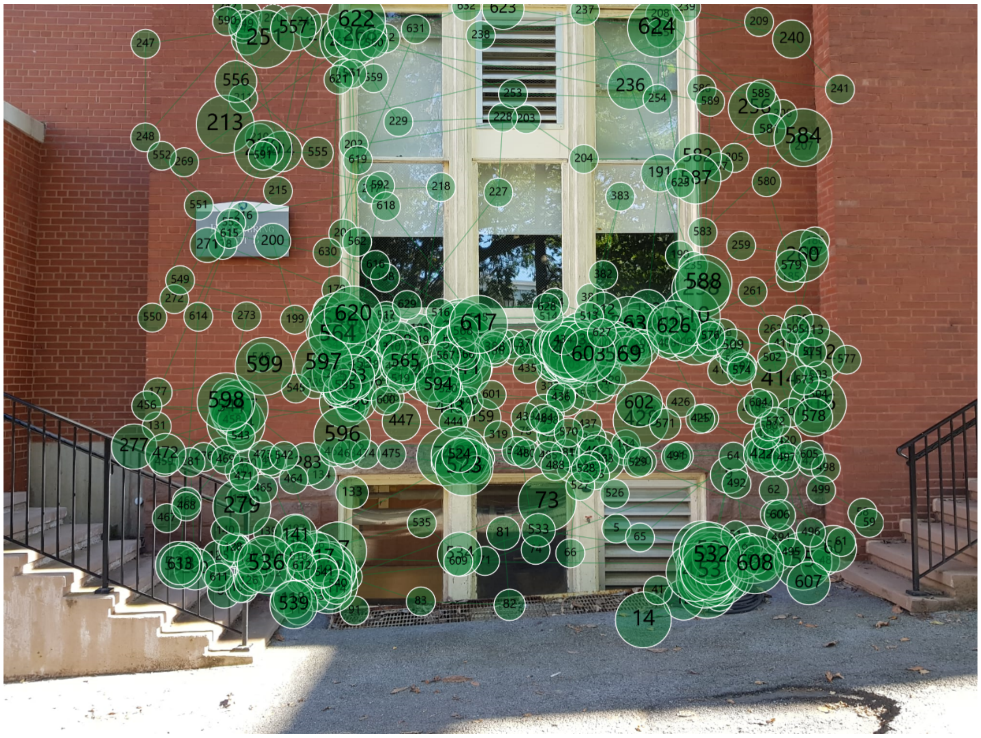

Supplementary Figure 39. Gaze plot of participant 8

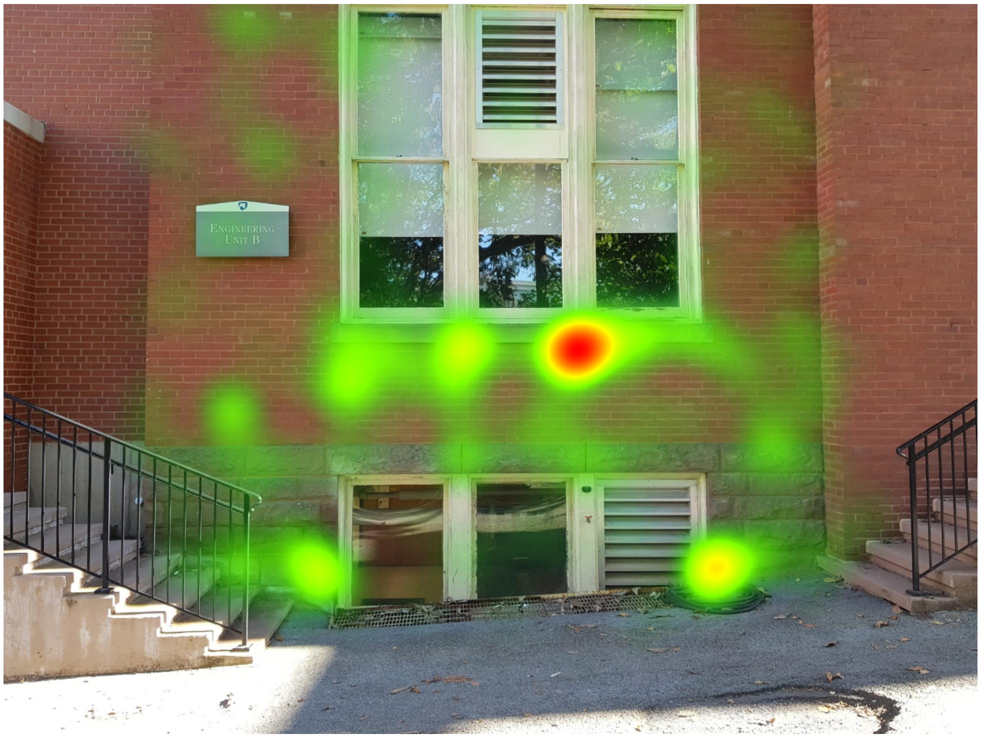

Supplementary Figure 40. Heat map of participant 8

Participant-9

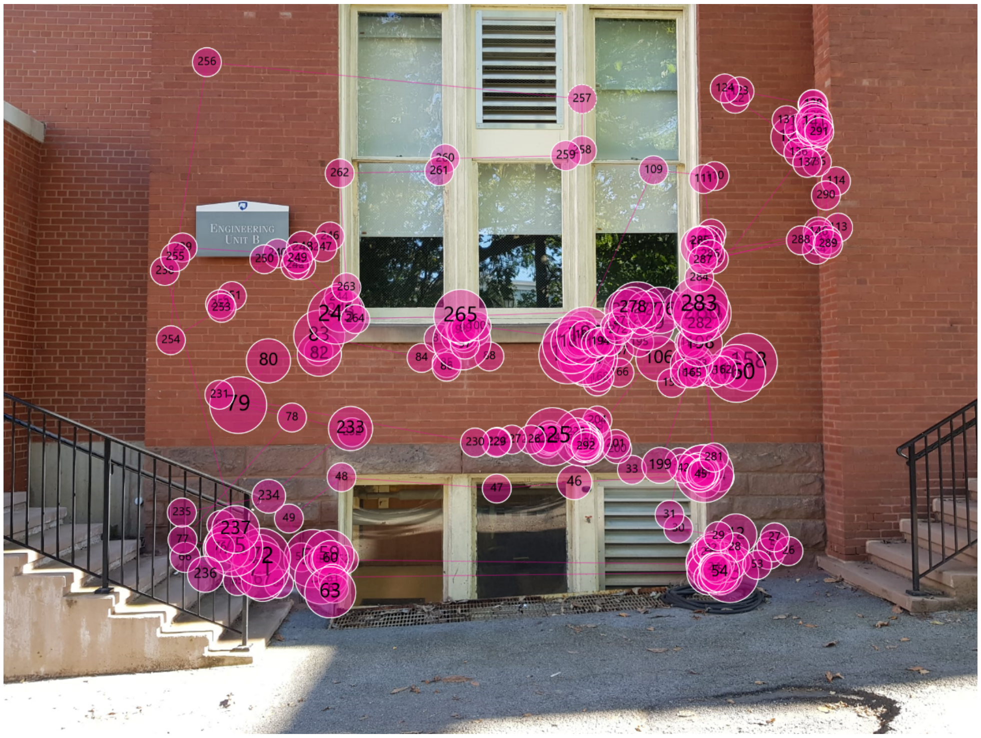

Supplementary Figure 41. Gaze plot of participant 9

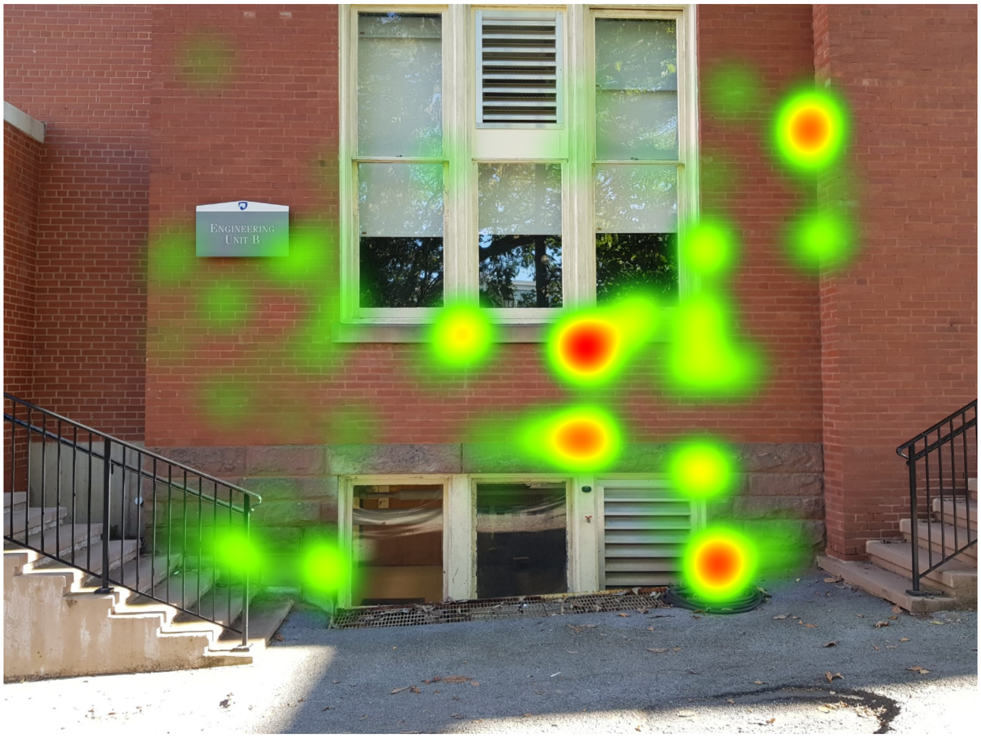

Supplementary Figure 42. Heat map of participant 9

Participant-10

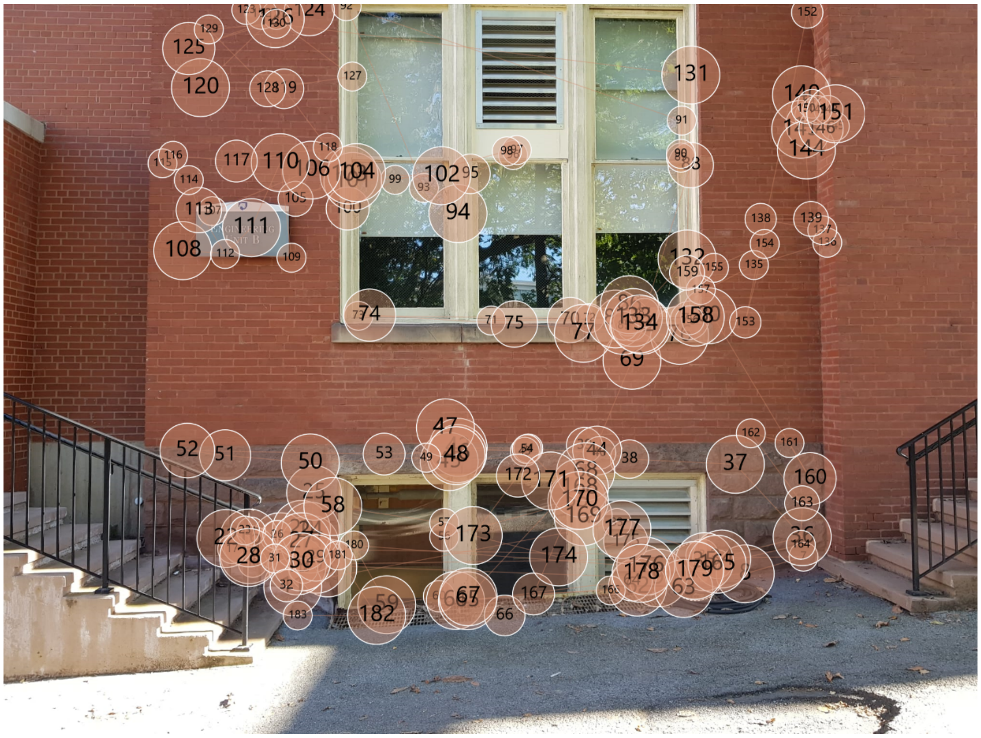

Supplementary Figure 43. Gaze plot of participant 10

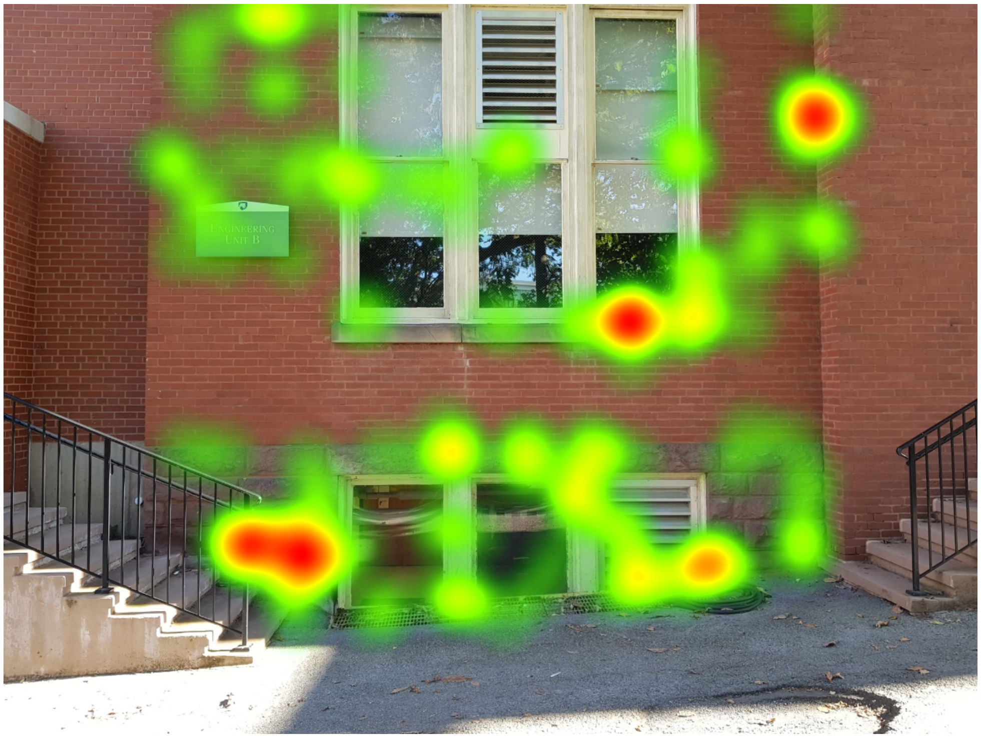

Supplementary Figure 44. Heat map of participant 10

Appendix VI

Table. 1 Statistical Analysis for different damage types

Fixation duration

	Building-1			Building-2		
	Mean diff.	t-value	p-value	Mean diff.	t-value	p-value
Crack	-1.179	16.07	<0.0001	-	-	-
Paint peel off	-0.1154	1.1111	>0.99	-	-	-
Biological growth	0.8397	5.667	0.0053	1.025	6.147	0.0019
Surface stain 1	1.004	6.818	0.0017	0.6318	3.568	0.0512
Surface stain 2	0.9931	5.937	0.0024	1.410	9.664	0.0002
Missing mortar 1	-	-	-	1.461	10.47	0.0003
Missing mortar 2	-	-	-	1.669	11.98	0.0001
Weathered stone 1	0.7936	6.583	0.0007	1.243	11.48	<0.0001
Weathered stone 2	-	-	-	1.176	8.061	0.0001

Fixation count

	Building-1			Building-2		
	Mean diff.	t-value	p-value	Mean diff.	t-value	p-value
Crack	1.027	16.08	<0.0001	-	-	-
Paint peel off	0.06244	0.5998	>0.9999	-	-	-
Biological growth	0.6449	5.680	0.0053	1.002	7.2	0.0006
Surface stain 1	0.8639	7.135	0.0013	0.7509	6.270	0.0017
Surface stain 2	0.9897	6.830	0.0009	1.391	13.95	<0.0001
Missing mortar 1	-	-	-	1.503	12.28	0.0001
Missing mortar 2	-	-	-	1.575	11.35	0.0002
Weathered stone 1	0.7308	6.966	0.0005	1.065	8.873	<0.0001
Weathered stone 2	-	-	-	0.9722	6.123	0.0012

Visit duration

	Building-1			Building-2		
	Mean diff.	t-value	p-value	Mean diff.	t-value	p-value
Crack	0.9694	17.34	<0.0001	-	-	-
Paint peel off	0.2978	2.149	0.4210	-	-	-
Biological growth	0.5768	3.693	0.0541	0.8590	4.87	0.0089
Surface stain 1	0.7410	4.691	0.0156	0.4505	2.452	0.2787
Surface stain 2	0.7746	4.412	0.0157	1.247	8.362	0.0005
Missing mortar 1	-	-	-	1.303	7.859	0.0016
Missing mortar 2	-	-	-	1.489	10.54	0.0003
Weathered stone 1	0.5666	4.819	0.0066	1.096	10.21	<0.0001
Weathered stone 2	-	-	-	1.004	6.887	0.0005

Visit count

	Building-1			Building-2		
	Mean diff.	t-value	p-value	Mean diff.	t-value	p-value
Crack	-	21.29	<0.0001	-	-	-
Paint peel off	-	8.420	0.0001	-	-	-
Biological growth	-	17.06	<0.0001	1.686	15.10	<0.0001
Surface stain 1	-	21.88	<0.0001	1.582	18.19	<0.0001
Surface stain 2	-	12.92	<0.0001	2.126	48.72	<0.0001
Missing mortar 1	-	-	-	2.374	30.86	<0.0001
Missing mortar 2	-	-	-	2.46	20.57	<0.0001
Weathered stone 1	-	27.24	<0.0001	1.668	24.19	<0.0001
Weathered stone 2	-	-	-	1.673	20.16	<0.0001

Note: For Building 1: weathered stone 1 refers to weathered stone; For Building 2: weathered stone 1 = weathered stone (left), and weathered stone 2 = weathered stone (right).
